# Rbm24 modulates adult skeletal muscle regeneration via regulation of alternative splicing

**DOI:** 10.7150/thno.44389

**Published:** 2020-09-11

**Authors:** Mengkai Zhang, Yue Han, Jing Liu, Lefeng Liu, Longqing Zheng, Yongxiong Chen, Rongmu Xia, Dongbo Yao, Xuemin Cai, Xiuqin Xu

**Affiliations:** 1 Institute of Stem Cell and Regenerative Medicine, School of Medicine, Xiamen University, Xiamen 361100, China.; 2 Shenzhen Research Institute of Xiamen University, Guangdong Province, 518000, P.R. China.; 3 Fujian Heze Biological Technology Co., Ltd, Xiamen, Fujian, China.; 4 Eye Institute of Xiamen University, Fujian Provincial Key Laboratory of Ophthalmology and Visual Science, Xiamen, Fujian, China.

**Keywords:** RNA binding motif protein 24, muscle injury, muscle regeneration, alternative splicing, myogenic differentiation

## Abstract

**Rationale:** The adult skeletal muscle can self-repair efficiently following mechanical or pathological damage due to its remarkable regenerative capacity. However, regulatory mechanisms underlying muscle regeneration are complicated and have not been fully elucidated. Alternative splicing (AS) is a major mechanism responsible for post-transcriptional regulation. Many aberrant AS events have been identified in patients with muscular dystrophy which is accompanied by abnormal muscle regeneration. However, little is known about the correlation between AS and muscle regeneration. It has been reported that RNA binding motif protein 24 (Rbm24), a tissue-specific splicing factor, is involved in embryo myogenesis while the role of Rbm24 in adult myogenesis (also called muscle regeneration) is poorly understood.

**Methods:** To investigate the role of Rbm24 in adult skeletal muscle, we generated Rbm24 conditional knockout mice and satellite cell-specific knockout mice. Furthermore, a cardiotoxin (CTX)-induced injury model was utilized to assess the effects of Rbm24 on skeletal muscle regeneration. Genome-wide RNA-Seq was performed to identify the changes in AS following loss of Rbm24.

**Results:** Rbm24 knockout mice displayed abnormal regeneration 4 months after tamoxifen treatment. Using RNA-Seq, we found that Rbm24 regulated a complex network of AS events involved in multiple biological processes, including myogenesis, muscle regeneration and muscle hypertrophy. Moreover, using a CTX-induced injury model, we showed that loss of Rbm24 in skeletal muscle resulted in myogenic fusion and differentiation defects and significantly delayed muscle regeneration. Furthermore, satellite cell-specific Rbm24 knockout mice recapitulated the defects in regeneration seen in the global Rbm24 knockout mice. Importantly, we demonstrated that Rbm24 regulated AS of Mef2d, Naca, Rock2 and Lrrfip1 which are essential for myogenic differentiation and muscle regeneration.

**Conclusions:** The present study demonstrated that Rbm24 regulates dynamic changes in AS and is essential for adult skeletal muscle regeneration.

## Introduction

Mechanical or pathological injuries of skeletal muscle are inevitable throughout an individual's life. Injured muscle is subjected to myofiber necrosis and muscle mass loss 1, leading to malfunctions of the muscle. Fortunately, adult skeletal muscle can autonomously repair these injuries. This regenerative capacity is due to the presence of muscle stem cells, also called satellite cells 2. Satellite cells express Pax7 quiescently. Upon damage, quiescent satellite cells are rapidly and extensively activated to generate Pax7^+^/Myod^+^ committed myogenic progenitor cells. Pax7^+^/Myod^+^ myoblasts further proliferate and differentiate to generate Myog^+^ fusion-competent myoblasts 3, 4. Myoblast fusion is a key step during skeletal muscle formation and repair 5. The differentiated myoblasts migrate and align appropriately to fuse with each other or with the damaged myofibers to produce multinuclear myofibers. Multinuclear myofibers undergo terminal differentiation and expand further in size, eventually forming functional myofibers indistinguishable from uninjured muscle 6. Although numerous studies have attempted to elucidate the complex and tightly regulated regeneration process 7-9, the hierarchical regulatory mechanisms of regeneration are still not fully understood.

Alternative splicing (AS) is a major mechanism responsible for generating multiple structurally and functionally different protein isoforms from a single gene. The effects of AS range from a complete loss of function to acquisition of a novel function 10, 11. AS often occurs in a cell or tissue type-specific manner and is instrumental for cell specification, tissue development and organ function maintenance 12. Dysregulation of AS can lead to severe malfunction and disease 13, 14. A considerable number of AS events have been identified during myogenesis in the past few years 15. In particular, a number of abnormal AS events have been identified in different types of muscular dystrophy, such as myotonic dystrophy types 1 and 2, both of which are accompanied by abnormal regeneration 16. However, to date, our knowledge regarding the role of AS in muscle regeneration is still limited. Thus far, only a few AS events, such as Mef2c, Mef2d and Naca, have been identified being spliced and involved in muscle regeneration regulation 17-19. Large efforts need to be done to clarify the AS effects on muscle regeneration.

RNA binding motif protein 24 (Rbm24) has been shown to function as a splicing factor responsible for muscle-specific AS 20. Our previous studies have reported that Rbm24 is expressed at the developing heart and somites during embryogenesis in vertebrates 21, 22. We also reported that Rbm24 regulates enormous AS events required for cardiac lineage differentiation and postnatal heart development 23, 24. Particularly, several previous studies have depicted the role of Rbm24 in embryonic myogenesis. It has been indicated that morpholino knockdown of Rbm24 significantly hinders somitogenesis and myogenic differentiation 25, 26. Additionally, Rbm24 promotes myogenic differentiation by stabilizing mRNA expression of myogenin during C2C12 (commonly referred to as myoblast) differentiation 27. There is also study demonstrating that Rbm24 is upregulated during mouse satellite cell-derived myoblast differentiation and inhibition of Rbm24 expression hinders the satellite cell-derived myoblast fusion process 28. These findings suggest that Rbm24 is essential for myogenesis during vertebrate development. However, to the best of our knowledge, there are no studies assessing the role of Rbm24 in muscle regeneration, and in particular, the effects of Rbm24-mediated AS on muscle regeneration.

In the present study, using a tamoxifen-inducible knockout strategy based on the Cre-loxP system, the role of Rbm24 in the regeneration of adult skeletal muscle in mice was examined. The present study is the first to show that Rbm24 positively regulates muscle regeneration by modulating myogenic fusion and differentiation. Furthermore, we showed that Rbm24 regulated a series of AS events, including AS of Mef2d, Naca, Lrrfip1 and Rock2, which are essential for myogenic differentiation and muscle regeneration.

## Materials and Methods

### Mice

All animal experiments were approved by the Institutional Animal Care and Use Committee of Xiamen University (Xiamen, Fujian, China; approval ID: SCXK2013-0006). The UBC-CreERT2 mice were purchased from Shanghai Model Organisms Center. The Pax7-CreER mice were provided by Dr. Dahai Zhu (Chinese Academy of Medical Sciences) and mdx mice were provided by Dr. Zhenji Gan (Model Animal Research Center of Nanjing University). The strategy for generation of Rbm24^loxP/loxP^ mice was described previously 24. To induce Rbm24 knockout, tamoxifen (MCE) dissolved in corn oil was injected intraperitoneally into 8-week-old mice at a daily dose of 0.1 g/kg body weight for 5 days. Additional details are provided in the Supplemental Methods.

### Muscle injury and regeneration

Mice were anesthetized throughout modeling using isoflurane. To assess muscle injury and regeneration, 2-month-old mice were used. Prior to inducing injury, the hind limb was disinfected using 75% alcohol. Then the tibialis anterior (TA) muscle of one limb was injected with 50 µl cardiotoxin (CTX) (10 μM; Sigma-Aldrich); and another limb was injected with an equivalent volume of PBS. The mice were subsequently placed in a new clean cage. For regeneration analysis, mice were sacrificed at different time points post-injury and bilateral TA muscles were dissected.

### Histological analysis

TA muscles were dissected from mice, embedded in the OCT compound (Tissue-Tek) and snap-frozen by immersing in liquid nitrogen. Using a cryostat, 10 μm cryosections were prepared (NX50; Thermo Fisher Scientific). The cryosections were stained with H&E according to the manufacturer's protocol (Nanjing Jiancheng) or were used for immunofluorescence staining for further analyses. Detailed information is shown in the Supplemental methods.

### Western blotting

For sample preparation, TA muscles were isolated, cut into pieces and then homogenized in RIPA lysis buffer. Supernatants were collected by centrifugation at 12000 rpm for 10 min at 4 °C and then boiled in loading buffer at 95 °C for 10 min. Proteins (25-40 μg) were separated in 12% SDS-PAGE gels and transferred to a 0.22 µm PVDF membrane (Pall Corporation). The PVDF membrane was blocked in 5% skimmed milk for 1 h at room temperature, and incubated with the primary antibody at 4 °C overnight. The HRP-conjugated secondary antibodies (Thermo Fisher Scientific) were incubated at room temperature for 1 h. The protein expression levels of Pax7, Myog, MF20 and Rbm24 were examined to determine the regeneration process. Additional details are provided in the Supplemental methods.

### RNA preparation and RNA-Seq

Samples were collected from hind limb TA muscles of 2-month-old male mice (n = 2) 3 days after tamoxifen administration. Total RNAs were extracted using TRIzol reagent according to the manufacturer's protocol (Invitrogen). RNA quality was assessed using agarose gel electrophoresis and by measuring the A260/A280 ratio to ensure integrity. Subsequently, the RNAs were pooled and sequenced using the Illumina HiSeq system (Illumina). Reads were aligned to the mouse transcriptome (GRCm38/mm10) using Tophat. Assembly of transcription was performed using Cufflinks 29. For differential gene expression, the DEseq package was used. AS was detected using rMATS and Astalavista 30, 31. Additional details are provided in the Supplemental methods.

### RT-PCR and qPCR

Total RNAs from cells or muscle tissue were prepared using TRIzol (Invitrogen) and cDNAs were synthesized using TransScript^®^ One-Step gDNA Removal and cDNA Synthesis SuperMix (TransGen Biotech) according to the manufacturer's protocol. PCR was performed using Taq DNA polymerase (Vazyme Biotech) according to manufacturer's protocol, with an annealing temperature of 58 °C (adjusted according to the primers) and 28-35 cycles for amplification. PCR products were stained with ethidium bromide and separated on a 1.5-3% agarose gel. qPCR was performed on an ABI 7500 (Applied Biosystems) using qPCR SYBR Green Mix (Vazyme Biotech) according to manufacturer's protocol, and a two-step method was used. For each 20 μL reaction, 1 μL 5 μM primer mix and 25 ng cDNA were added. Primer sequences for all PCRs performed are listed in Table S6. Details of alternative exon positions in each gel image in Figures are listed in Table S7.

### *In vitro* splicing assay

For minigene construction, mouse genomic DNAs were extracted from TA muscle using a TIANamp Genomic DNA kit (TIANGEN Biotech). The corresponding mouse genomic DNA fragments were amplified from mouse genomic DNA using high-fidelity polymerase (TransGen Biotech) with *Kpn*I and *Xho*I restriction enzyme sites flanking at the end. Subsequently, the fragments were purified, recycled and cloned into pcDNA3.1 (+) plasmid using EasyGeno Assembly Cloning kit (TIANGEN Biotech). Similarly, the human Rbm24 coding sequence was amplified with the PCR primers containing *Xho*I and *Kpn*I restriction enzyme sequences and were cloned into the pXJ40 vector 32. Plasmids expressing Rbm24 and the minigene (1:1) were transfected into 293T cells using Lipofectamine 3000 (Invitrogen). After 48 h of transfection, total RNAs were extracted and reverse transcribed. Splicing assay was performed by RT-PCR. The sequences of the primers used are provided in Table S6.

### Cell culture and differentiation

293T and C2C12 cells were cultured in High- Glucose DMEM (HyClone) supplemented with 10% FBS (HyClone) and 1% penicillin/streptomycin. For C2C12 differentiation, C2C12 cells were transferred into differentiation medium supplemented with 2% horse serum when the cells were >90% confluent. Differentiated cells were collected at 3 days after differentiation for further analyses.

### Cell transfection and transduction

293T cells were transfected using Lipofectamine 3000 (Invitrogen) according to the manufacturer's protocol, when the cells were 60% confluent. For Rbm24 knockdown, undifferentiated C2C12 cells were transduced with lentiviruses expressing non-targeting shRNA (shCTL) or shRNA directed against Rbm24 (shRbm24) with 8 μg/mL polybrene. After 48 h of transduction, cells were selected using 1 mg/mL G418 for 2 days. Rbm24 knockdown C2C12 cells underwent another two passages before the rescue experiments were performed. For rescue experiments, Rbm24 knockdown C2C12 cells were transduced with another lentivirus expressing either a flag-tagged undifferentiated isoform Mef2dα1 or flag-tagged differentiated isoform Mef2dα2 with 8 μg/mL polybrene and then transfected cells were selected using 2 μg/mL puromycin for another 2 days before differentiation.

## Results

### Rbm24 knockout mice show abnormal muscle regeneration

**Figure 1:**
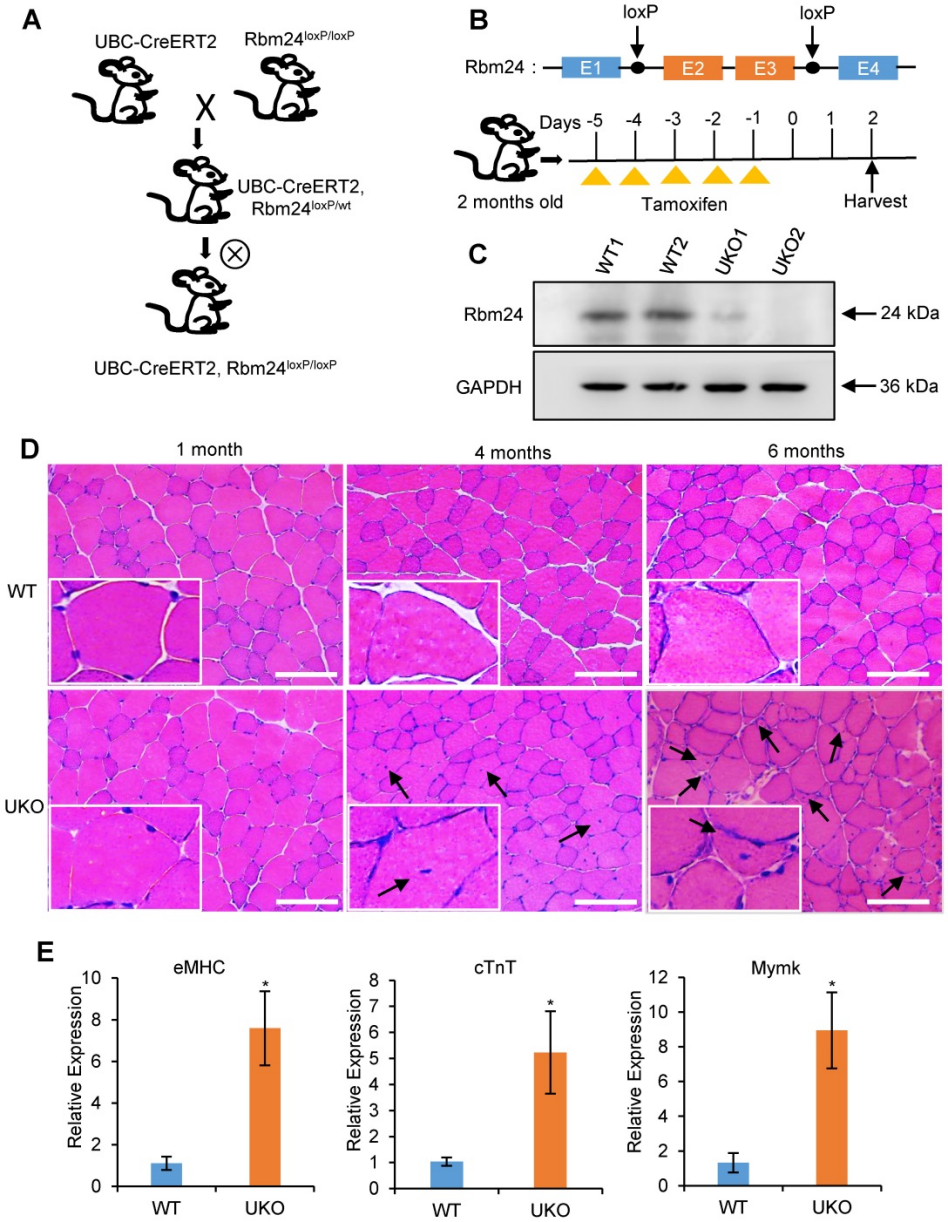
** Abnormal muscle regeneration in Rbm24 knockout mice.** (**A**) Schematic representation of the construction of the UKO mice. We generated UKO mice by breeding Rbm24^loxP/loxP^ mice with UBC-CreERT2 mice. (**B**) Schematic showing the generation of Rbm24 conditional alleles and the method of drug administration. Exons 2 and 3 of Rbm24 were flanked by two loxP sites. The loxP sites are indicated by the arrowheads. Rbm24 deletion was induced by injection of tamoxifen intraperitoneally for 5 consecutive days in 8-week-old mice. (**C**) Western blot analysis of Rbm24 knockout efficiency in skeletal muscle from 8-week-old mice 3 days after tamoxifen treatment, GAPDH was used as a loading control. Two samples from each group were used as representative examples. (**D**) H&E staining showing the abnormal regeneration of muscle in mice 4 to 6 months after tamoxifen treatment. Regenerated myofibers with centralized nuclei are denoted by arrowheads. Scale bars, 100 µm. (**E**) Quantification of the regeneration markers using qPCR. The elevated expression of eMHC, cTnT and Mymk indicated the abnormal regeneration of muscle in the UKO mice. Total RNAs were extracted from the skeletal muscle at 6 months after tamoxifen treatment. Data are presented as mean ± SEM, n = 4, *P < 0.05, unpaired *t*-test. Abbreviations: cTnT: cardiac Troponin T; eMHC: embryonic MHC; Mymk: myomaker; qPCR: quantitative real-time polymerase chain reaction; UKO: UBC-CreERT2, Rbm24^loxP/loxP^.

To investigate the role of Rbm24 in adult skeletal muscle, we generated the Rbm24 conditional knockout mice termed UBC-CreERT2, Rbm24^loxP/loxP^ (UKO) mice, in which Rbm24 could be globally disrupted by injection of tamoxifen (Figure 1A). The scheme of construction of the Rbm24 allele and drug administration are shown in Figure 1B. Rbm24 was efficiently deleted in the skeletal muscle 3 days after tamoxifen injection (Figure 1C).

UKO mice exhibited a slightly decreased body weight and muscle mass in the first 2 months post tamoxifen injection. We also observed a reduction in muscle strength 3-4 months later (Figure S1). Histological analyses indicated that the skeletal muscle from UKO mice was indistinguishable from those of the wild type (WT) littermates 1 month after tamoxifen injection, as was indicated by the uniform and intact arrangement of myofibers (Figure 1D, left panel). However, 4 months after tamoxifen administration, the number of myofibers with centralized myonuclei increased in the UKO mice, which is indicative of regeneration, whereas this was not observed in the WT mice (Figure 1D, middle panel). Furthermore, there were more myofibers with smaller sizes and centralized myonuclei after 6 months in the UKO mice, which indicated newly formed myofibers were present (Figure 1D, right panel). These observations suggest the occurrence of abnormal regeneration in the UKO mice. Abnormal regeneration was further confirmed by the elevated expression of the regeneration-related markers (Figure 1E), such as embryonic MHC (eMHC), cardiac troponin T (cTnT) and myomaker (Mymk) 33-36. Together, these results indicate that UKO mice undergo abnormal muscle regeneration.

### Rbm24 expression is elevated during regeneration

**Figure 2:**
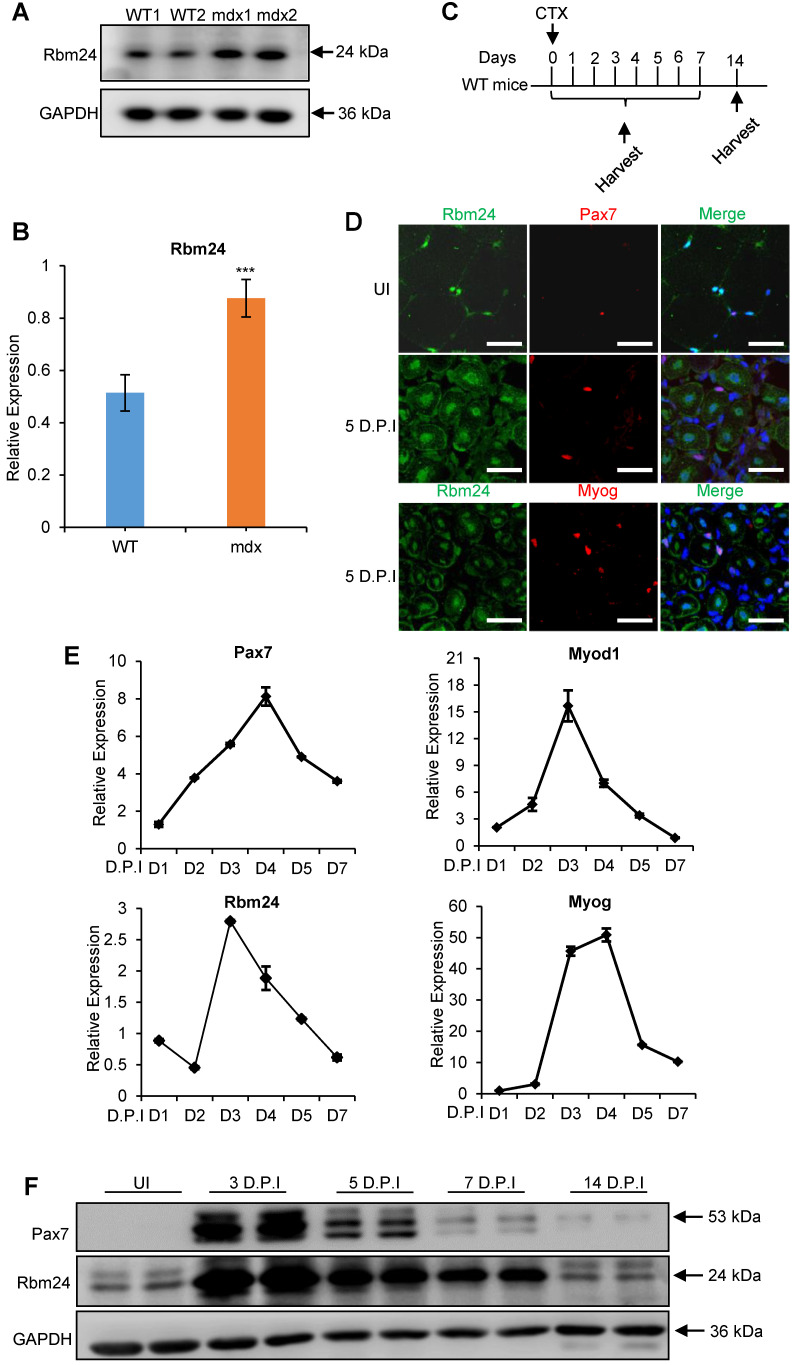
** Elevated Rbm24 expression during skeletal muscle regeneration.** (**A**) Rbm24 expression was upregulated in the TA muscle of mdx mice compared with the WT mice of the same age. The 4-week-old mdx mice were used for the pathological muscle regeneration model. For each group, 2 samples were used as representatives. GAPDH was used as a loading control. (**B**) Histogram showing the expression levels of Rbm24 in (A), Rbm24 was normalized to GAPDH. Data are presented as mean ± SEM, n = 4, ***P < 0.001, unpaired *t*-test. (**C**) Schematic diagram depicting CTX-induced injury and specimen collection. TA muscle of 2-month-old WT mice was injured by CTX injection and samples were collected at the indicated time points. (**D**) Immunostaining analysis of Rbm24 expression in uninjured and injured TA muscle. TA muscle of 2-month-old WT mice was used for analysis of the localization of Rbm24 before and after injury. Muscle injury was induced by injection of CTX. After 5 D.P.I, TA muscle was collected for analysis. Scale bars, 20 µm. (**E**) Expression profiles of Rbm24 and regeneration markers (Pax7, Myod1 and Myog) during skeletal muscle regeneration. Data are presented as mean ± SEM, n = 3. (**F**) Western blot analysis of Rbm24 expression during muscle regeneration. GAPDH was used as the loading control. Abbreviations: CTX: cardiotoxin; D.P.I: days post injury; mdx: X-linked muscular dystrophy; TA muscle: tibialis anterior muscle; UI: uninjured; WT: wild type.

Whether Rbm24 is involved in muscle regeneration was next determined. Rbm24 expression in the muscle regeneration model was assessed. Mdx mice are widely used to study muscular dystrophy, and undergo abnormal muscle regeneration during postnatal development 37-39. We first examined Rbm24 expression in the mdx mice. According to previous studies, the tibialis anterior (TA) muscle of mdx mice do not exhibit signs of regeneration during the first 2 weeks after birth, and extensive regeneration is seen in 4-week-old mdx mice, after which, regeneration activity is decreased 40, 41. Interestingly, we found that Rbm24 expression was increased in the TA muscle of 4-week-old mdx mice compared with the WT mice, but not in the 2-week-old mdx mice (Figures 2A-B, S2A-B). The increased expression of Rbm24 decreased as the mdx mice developed. Thus, Rbm24 expression mimics the regeneration activity of mdx mice, which suggests the potential role of Rbm24 in regulation of the muscle regeneration of mdx mice.

Additionally, we determined whether Rbm24 participated in muscle regeneration by assessing Rbm24 expression during regeneration in the CTX-induced muscle injury and regeneration model. Acute muscle injury was induced by injection of CTX into the TA muscle 42. Details of injury induction and sample collection are described in Figure 2C. As adult skeletal muscles can regenerate effectively primarily due to the presence of satellite cells, we determined the expression levels of Rbm24 in the satellite cells before and after injury. Immunofluorescence staining results indicated that Rbm24 was primarily localized at the peripheral myonuclei when the muscle was uninjured (UI) and was barely detectable in the quiescent satellite cells as shown in the top panel of Figure 2D. Similarly, there was only a low amount of Rbm24 expression observed in the activated satellite cells 5 days post injury (D.P.I) (Figure 2D middle panel). In fact, the expression of Rbm24 in Myog^+^ cells was relatively high, except in the centralized myonuclei in the injured muscle (Figure 2D, bottom panel), which suggests that Rbm24 may be involved in the myogenic differentiation process during muscle regeneration. These results were consistent with the published RNA-Seq data of satellite cells (Figure S2C-D) 43.

We also analyzed the dynamic expression of Rbm24 during regeneration following injury (Figure 2E). The expression of Pax7 and Myod1 were rapidly upregulated in the first 2 D.P.I, reflecting the activation of satellite cells, which is a sign of initiation of regeneration 4. Meanwhile, Rbm24 expression was downregulated at 2 D.P.I, possibly due to the necrosis of myofibers. At 3 D.P.I, the expression levels of both Myod1 and Myog were markedly elevated, indicating the transition to the myoblast fusion and differentiation stage. Similarly, Rbm24 expression was also rapidly upregulated at 3 D.P.I, suggesting that Rbm24 may be involved in the differentiation process during regeneration. At 5 D.P.I, the expression levels of Pax7, Myod1, Myog and Rbm24 were gradually restored to normal levels. The dynamic expression of Rbm24 during regeneration was also validated by western blot analysis (Figure 2F). Together, these findings suggest that Rbm24 is involved in skeletal muscle regeneration.

### Loss of Rbm24 impairs muscle regeneration following injury

**Figure 3:**
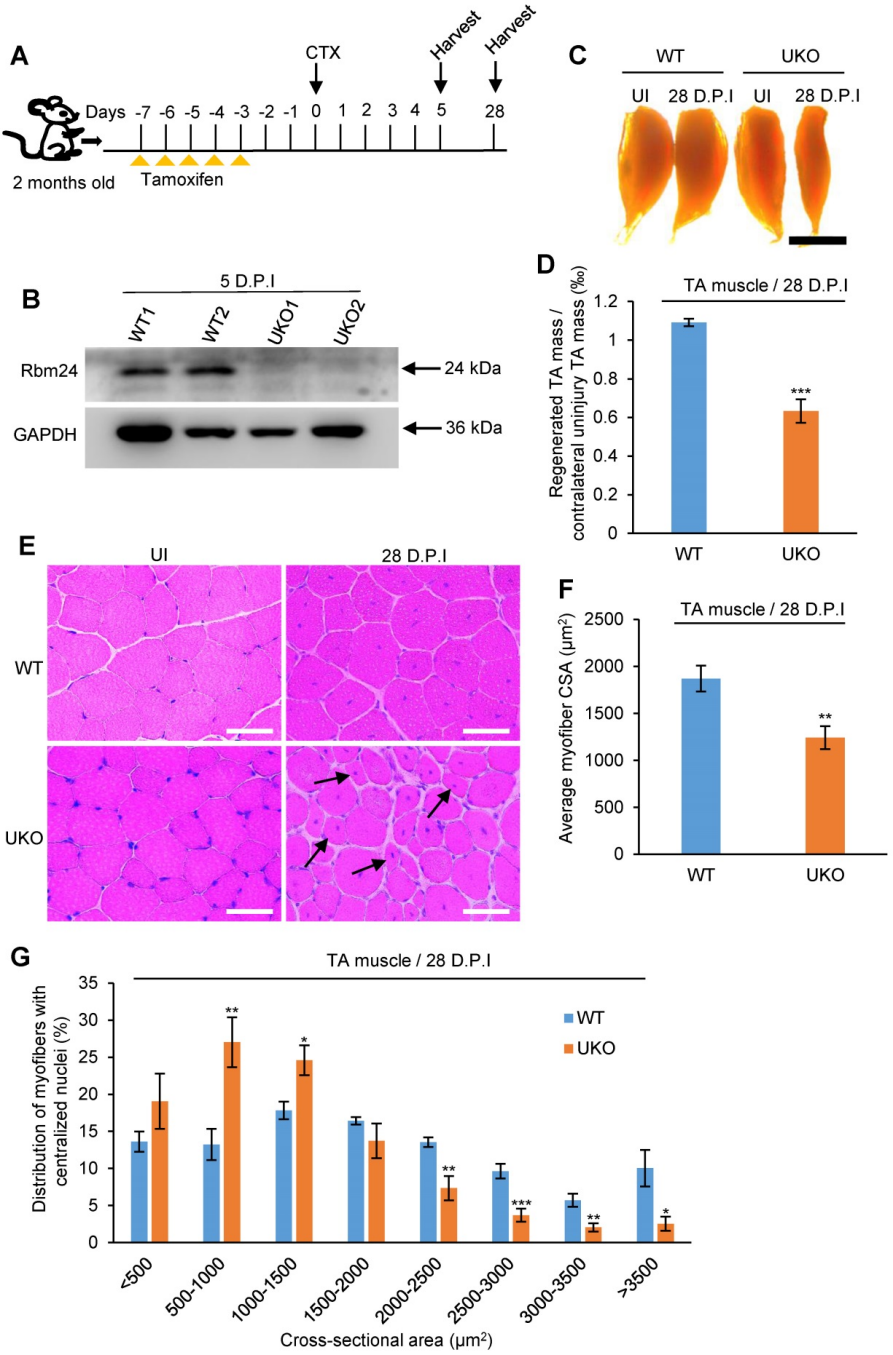
** Inducible deletion of Rbm24 delays skeletal muscle regeneration.** (**A**) Schematic representation of CTX-induced injury and tamoxifen administration. The TA muscle was harvested at the indicated time points following injury. (**B**) Validation of Rbm24 knockout in the injured TA muscle at 5 D.P.I using western blotting. GAPDH was used as a loading control. (**C**) Gross morphology analysis of regenerated TA muscle at 28 D.P.I. The smaller regenerated muscle mass in UKO mice showed the delayed regeneration process. Scale bars, 2 mm. (**D**) Statistical analysis of regenerated TA muscle mass (n = 7 per group). (**E**) H&E staining showing deficient regeneration in the UKO mice. The increased myofibers with smaller sizes in the UKO mice were indicated by the arrowheads. Scale bars, 50 µm. (**F**) Statistical analysis of the average CSA of regenerated myofibers with centrally located nuclei. The CSA of the regenerated myofibers was significantly decreased in the UKO mice. At least 1,200 myofibers were analyzed in each mouse (n = 7 per group). (**G**) Distribution of regenerated myofibers with different CSA. There was a striking left shift in the UKO mice compared with the WT mice. At least 1,200 myofibers were analyzed in each mouse (n = 7 per group). Data are presented as the mean ± SEM. *P < 0.05, **P < 0.01, ***P < 0.001; unpaired t-test. Abbreviations: CSA: cross-sectional area; CTX: cardiotoxin; D.P.I: days post injury; NS: not significant; TA muscle: tibialis anterior muscle; UKO: UBC-CreERT2, Rbm24^loxP/loxP^; WT: wild type.

We further explored the effects of Rbm24 on muscle regeneration in Rbm24 knockout mice. Muscle damage was induced by injection of CTX into the TA muscle of 2- month-old mice 3 days after tamoxifen treatment. The scheme of drug administration and sample collection is shown in Figure 3A. Rbm24 knockout efficiency was evaluated by western blot analysis (Figure 3B).

To compare the regeneration capacity, TA muscle was dissected at 28 D.P.I. The regenerated muscle in UKO mice was notably smaller than the contralateral uninjured muscle, whereas the injured TA muscle in the WT group was effectively repaired and comparable to the contralateral uninjured muscle (Figure 3C), suggesting impaired regeneration capacity in the UKO mice. Consistent with the gross examination, the weight of regenerated TA muscle in the UKO mice was also significantly reduced (Figure 3D).

Histological analysis indicated that at 28 D.P.I, WT mice had regenerated muscle fibers with a normal size, whereas in the UKO mice, there were still myofibers with smaller diameters (Figure 3E). We also found that the average cross-sectional area (CSA) of the regenerated myofibers in the UKO mice was significantly reduced (Figure 3F). Further analysis of myofiber size distribution showed a noticeable left shift in the UKO mice, which indicated that overall, the regenerated fibers were smaller (Figure 3G). Collectively, these findings indicated that the loss of Rbm24 impairs skeletal muscle regeneration.

### Rbm24 is required for myogenic fusion and differentiation during muscle regeneration

During skeletal muscle development, muscle growth largely occurs due to fusion of myoblasts 5. As regenerated myofibers in UKO mice were significantly smaller, whether Rbm24 disruption interrupted the myoblast fusion and differentiation process during regeneration needs to be determined. Considering that muscle cell differentiation primarily occurred at 5-7 D.P.I 44, we analyzed early regeneration in the UKO mice at 5 D.P.I.

**Figure 4:**
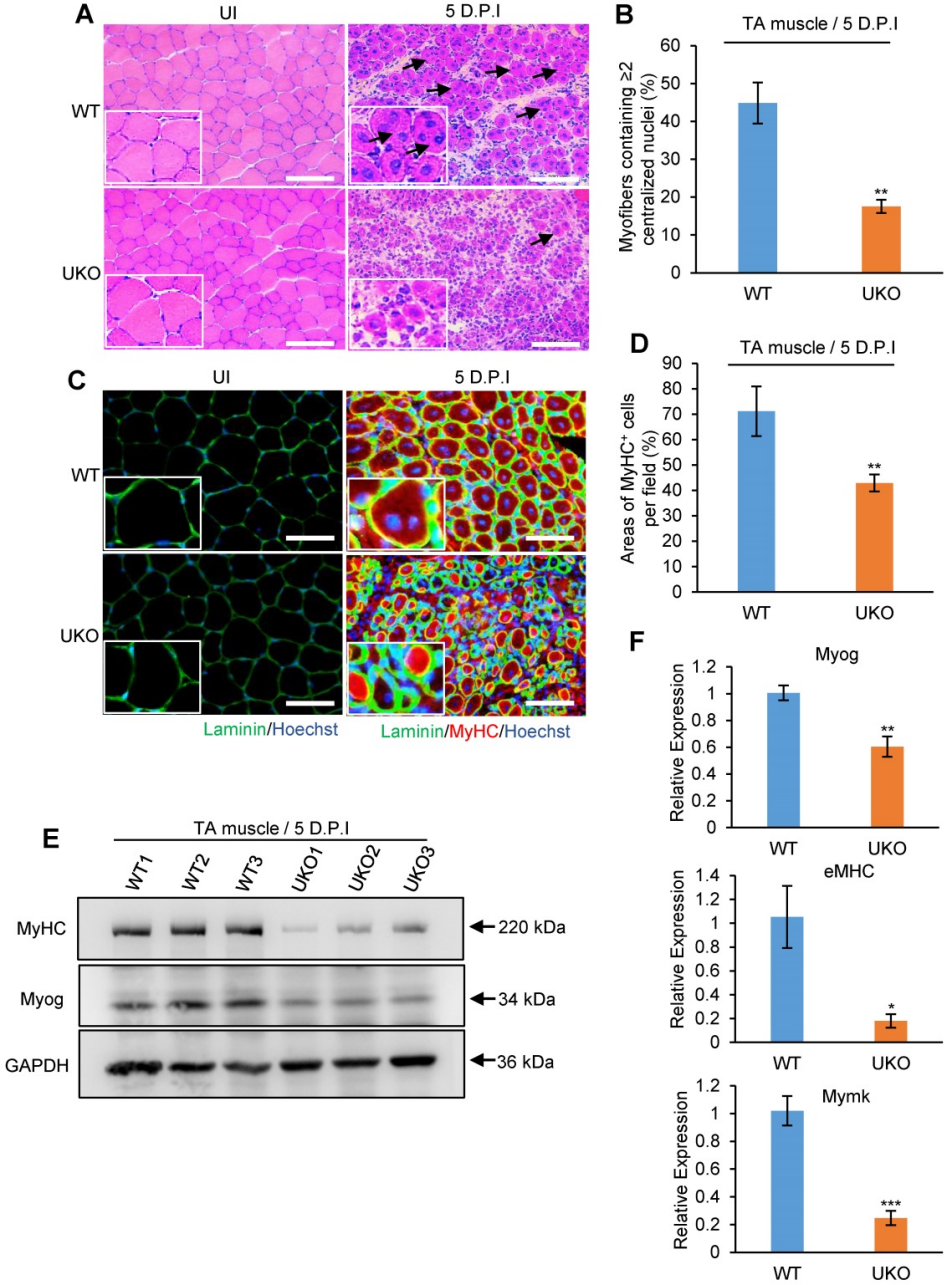
** Loss of Rbm24 impairs the myogenic process during regeneration.** (**A**) H&E staining showing fusion defects during regeneration at 5 D.P.I in the UKO mice. The well-formed myofibers with more than two centralized nuclei are indicated by arrowheads. Scale bars, 100 µm. (**B**) Statistical analysis of the number of regenerated myofibers containing two or more centralized nuclei per field. At least 250 myofibers were analyzed in each mouse, n = 6 per group. (**C**) Immunofluorescence analysis of MyHC-positive fibers in the regenerated TA muscles. The decreased MyHC-positive area is indicative of differentiation defects during regeneration in the UKO mice. Scale bars, 50 µm. (**D**) Statistical analysis of the MyHC-positive area per field in regenerated TA muscle (n = 4 per group). (**E**) Western blot analysis of MyHC and Myog expression in regenerated TA muscle. GAPDH was used as the loading control. The decreased expression of terminal differentiation markers MyHC and Myog validated the impaired differentiation capacity in the UKO mice. (**F**) Expression analysis of Myog, eMHC and Mymk during regeneration. Decreased mRNA expression levels of Myog, eMHC and Mymk were further indicative of deficient myogenic fusion and differentiation process in the UKO mice. Total RNAs were extracted from the regenerated TA muscle at 5 D.P.I. N = 4 in each group. Data are presented as mean ± SEM, *P < 0.05, **P < 0.01, ***P < 0.001, unpaired *t*-test. Abbreviations: D.P.I: days post injury; eMHC: embryonic MyHC; Mymk: Myomaker; TA muscle: tibialis anterior muscle; UKO: UBC-CreERT2, Rbm24^loxP/loxP^.

H&E staining revealed that WT mice possessed efficiently regenerated fibers, with a sharp outline, larger CSA and uniform size at 5 D.P.I. However, the majority of nascent fibers exhibited notably smaller diameters and it was not possible to detect the location of the nuclei in some of these fibers in the UKO mice (Figure 4A), which further highlights the defective regeneration. Importantly, we found a significant decrease in the number of myofibers containing more than two centralized myonuclei in the UKO mice, indicative of a reduced fusion index (Figure 4B). Additionally, the number of MyHC-positive myofibers in the UKO mice also significantly decreased, which provides evidence of defective differentiation (Figure 4C-D). This result was further confirmed by the insufficient expression of Myog and MyHC proteins observed (Figure 4E). During myogenesis, Mymk is transiently expressed and indispensable for myoblast fusion 45. eMHC is a hallmark of muscle regeneration and required for skeletal muscle differentiation 46, 47. Consistent with defective myoblast fusion and differentiation during regeneration, the mRNA expression levels of Myog, eMHC and Mymk were downregulated in the UKO mice (Figure 4F). In summary, these results suggest that loss of Rbm24 leads to myoblast fusion and differentiation defects during muscle regeneration.

### Specific deletion of Rbm24 in satellite cells recapitulates the regeneration defects

**Figure 5:**
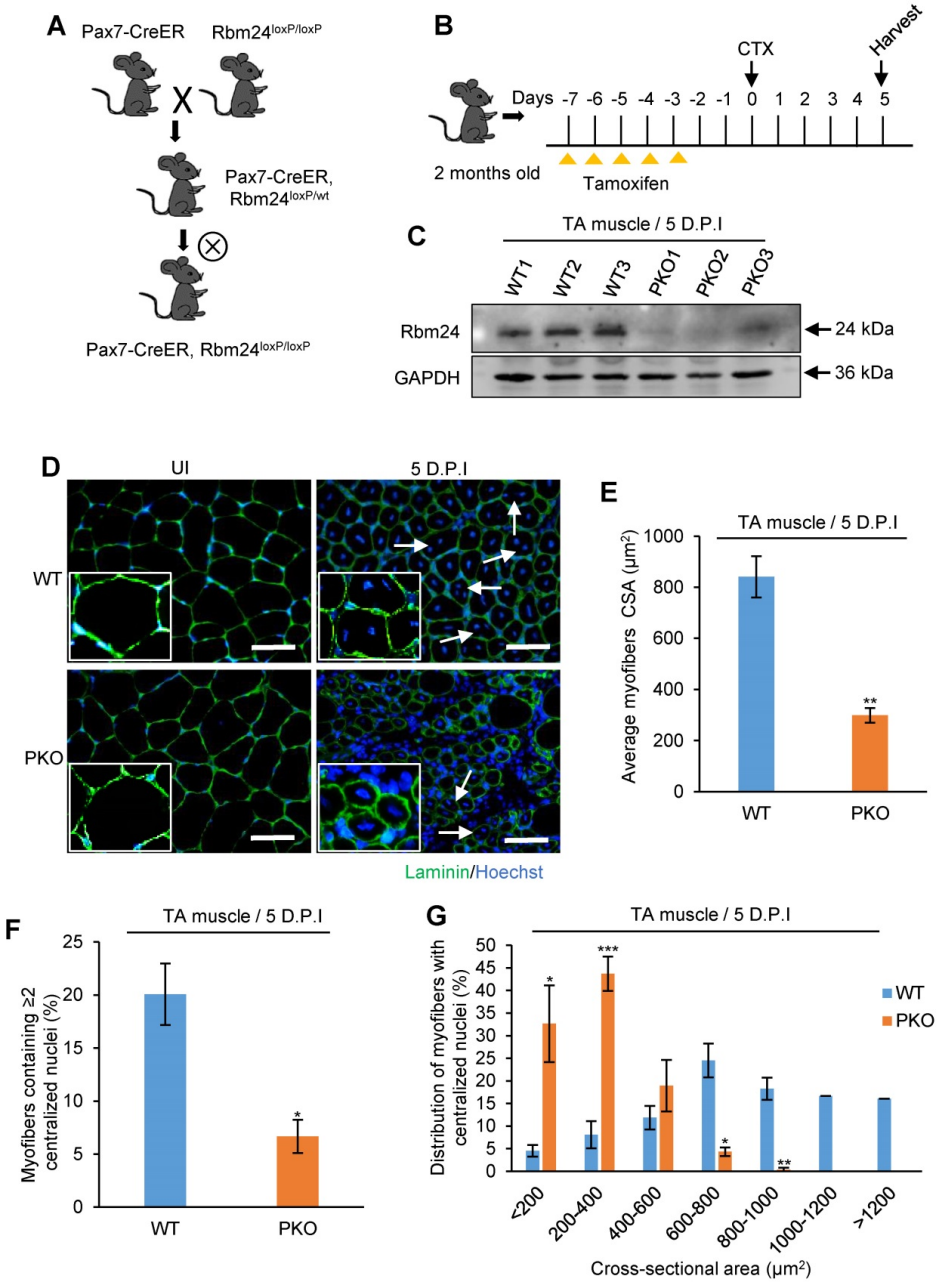
** Specific deletion of Rbm24 in satellite cells hinders skeletal muscle regeneration.** (**A and B**) Scheme of generation of PKO mice (A) and drug administration (B). (**C**) Western blot analysis to assess Rbm24 knockout efficiency in the injured TA muscle of PKO mice. TA muscle was dissected for sample preparation at 5 D.P.I. GAPDH was used as a loading control. (**D**) Immunostaining showing the regeneration defects at 5 D.P.I in the PKO mice. The well-formed myofibers with more than two centrally located nuclei and larger sizes are indicated by the arrowheads. Scale bars, 50 µm. (**E**) Average CSA of regenerated myofibers. At least 100 myofibers were analyzed in each mouse (n = 4-5 per group). (**F**) The number of myofibers containing two or more centralized myonuclei per field at 5 D.P.I. At least 100 myofibers were analyzed in each mouse (n = 4 per group). (**G**) Distribution of regenerated myofibers with different sizes at 5 D.P.I. At least 100 myofibers were analyzed in each mouse (n = 4-5 per group). Data are presented as the mean ± SEM. *P < 0.05, **P < 0.01, ***P < 0.001, unpaired *t*-test. Abbreviations: CSA: cross-sectional area; D.P.I: days post injury; PKO: Pax7-CreER, Rbm24^loxP/loxP^; TA muscle: tibialis anterior muscle.

Adult skeletal muscle retains its regeneration capacity primarily due to the function of satellite cells. To avoid the potential compensatory effects in UKO mice on skeletal muscle regeneration, we generated Pax7-CreER, Rbm24^loxP/loxP^ (PKO) mice, in which specific deletion of Rbm24 in satellite cells can be achieved (Figure 5A). The drug administration scheme is presented in Figure 5B. Rbm24 was efficiently deleted in the PKO mice at 5 D.P.I (Figure 5C).

To determine whether PKO mice exhibited a similar phenotype to the UKO mice, we analyzed regeneration at 5 D.P.I. Our results indicated that there was an increased number of smaller myofibers and there were more blank areas in the PKO mice. By contrast, the regenerated muscle of WT mice primarily consisted of fibers with larger diameters and the damaged myofibers were largely repaired (Figure 5D). Additionally, the average CSA of nascent muscle fibers (Figure 5E) and the number of myofibers containing two or more centralized myonuclei (Figure 5F) significantly reduced in the PKO mice compared with the control group. Furthermore, a similar left shift of fiber size distribution was also found in the PKO mice (Figure 5G). Overall, these results in the PKO mice successfully recapitulated the phenotype observed with the UKO mice, which together demonstrate that Rbm24 regulates muscle regeneration by regulating satellite cell-derived myoblast fusion and differentiation.

### Rbm24 deficiency results in transcriptomic changes in adult skeletal muscle

**Figure 6:**
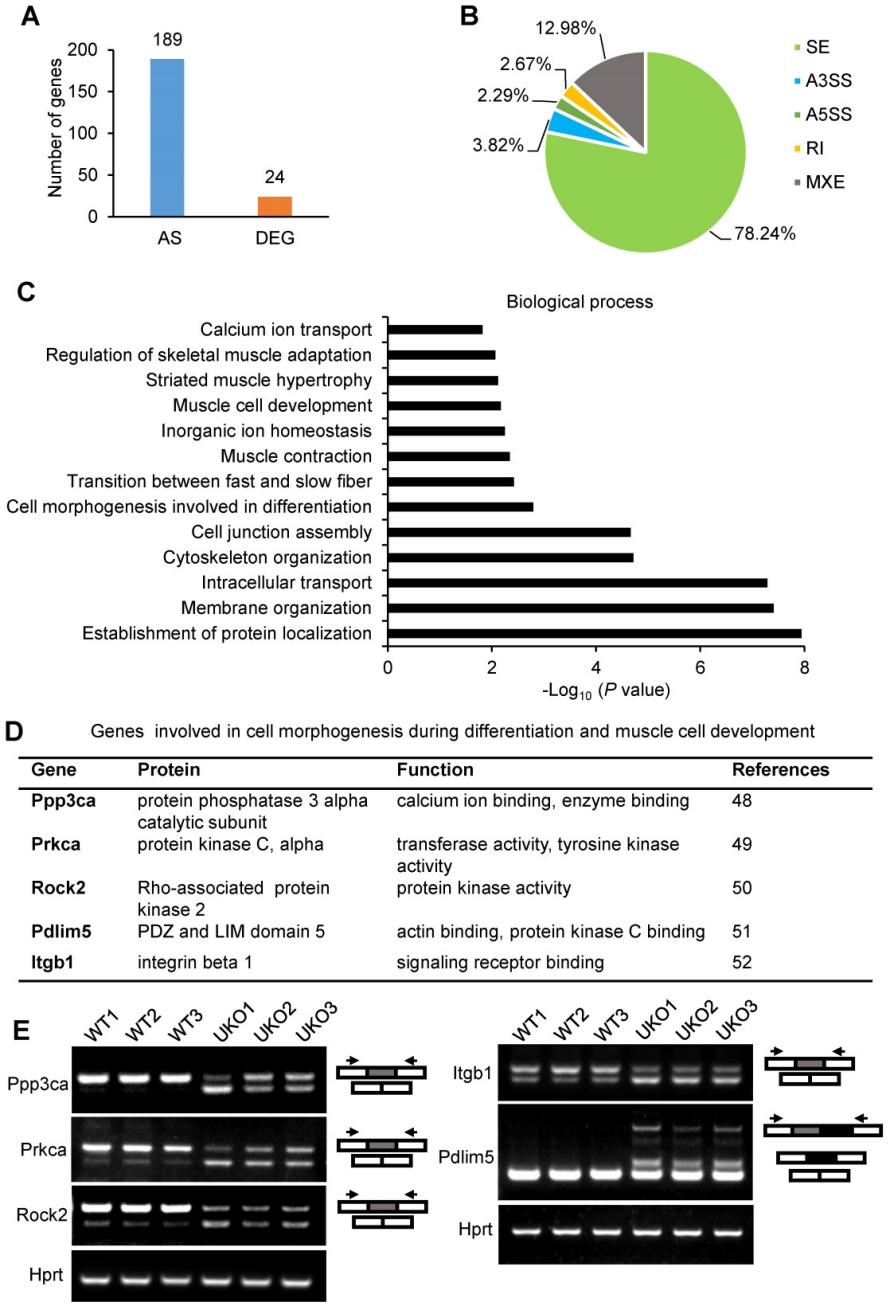
** Transcriptome remodeling in the Rbm24 knockout mice.** (**A**) Bar chart showing the number of DEGs and genes that underwent AS in Rbm24 knockout mice. (**B**) Pie chart showing the distribution of different types of AS events. (**C**) GO analysis of genes that were alternatively spliced in response to loss of Rbm24. Genes that were involved in cell differentiation, muscle cell development and muscle hypertrophy were significantly enriched. (**D**) A table showing the genes that were alternatively spliced by Rbm24 and involved in cell morphogenesis during differentiation and muscle cell development. (**E**) RT-PCR analysis to validate the AS of genes mentioned in (D). Total RNAs were extracted from intact TA muscle 3 days after tamoxifen treatment. The primers used for RT-PCR are indicated by the arrows. Hprt was used as the loading control. White box, flanking constitutive exons; gray and black box, alternative exons. Abbreviations: AS: alternative splicing; A5SS, A3SS: alternative 5' and 3' splice sites; DEG: differential expression genes; GO: Gene Ontology; MXE: mutually exclusive exons; RI: retained exons; RT-PCR: reverse transcription-polymerase chain reaction; SE: skipped exons.

We next sought to uncover the mechanism underlying the abnormal regeneration of UKO mice. Total RNAs were extracted from TA muscle for RNA-Seq 3 days after tamoxifen treatment. The sample information is listed in Table S1 and a panel of candidate genes identified by the RNA-Seq analysis were validated in Figure S3A and B. Each sample produced 39-54 million paired-end reads, of which 82-84% mapped uniquely to the mouse genome (Table S1). The RNA-Seq results indicated that loss of Rbm24 had only a relatively small effect on gene expression 3 days after tamoxifen treatment in uninjured TA muscle; only 24 genes exhibited a ≥2-fold differential expression (Table S2). By contrast, 189 genes underwent AS (Figure 6A). The AS events are listed in Table S3. Consistent with our previous study 24, Rbm24 regulated several types of AS events, including mutually exclusion exon (MXE), retained intron (RI), skipped exon (SE) and alternative 5' and 3' splice sites (A5SS and A3SS), and the most prominently affected type of AS was SE (78.24%) (Figure 6B). Additionally, genes that were differentially expressed and alternatively spliced showed no overlapping. We also compared our results with the published transcriptome data from Rbm24 knockout mice hearts (Figure S3F). By comparison, we found Rbm24 regulated a series of common splicing events in the heart and skeletal muscle, which suggested conservation of the function of these AS events in the muscle system. Importantly, we found that loss of Rbm24 resulted in a large number of abnormal AS events in skeletal muscle that did not occur in the heart, highlighting the potential specific role of these AS events in skeletal muscle.

Next, we focused on analysis of genes that were alternatively spliced. Gene Ontology (GO) analysis showed a significant enrichment of genes involved in cell morphogenesis during differentiation, muscle cell development, muscle contraction and muscle homeostasis (Figure 6C). KEGG pathway analysis also identified significant enrichment of pathways, such as the calcium signaling pathway, cGMP-PKG signaling pathway and Wnt signaling pathway, which are indispensable for muscle development (Figure S3D). Thus, Rbm24 regulated AS of genes involved in multiple aspects, which are necessary for muscle function.

Considering the defects of myogenic differentiation during regeneration in Rbm24 KO mice, we next focused on the AS analyses of genes involved in cell morphogenesis during differentiation and muscle cell development. Based on the GO analyses, Ppp3ca, Prkca, Rock2, Pdlim5 and Itgb1 were further analyzed (Figure 6D). Ppp3ca exhibits different expression patterns during goat muscle development, indicating its potential function in the muscle 48. Prkca can induce ventricular myocyte hypertrophy in neonatal rats 49. During C2C12 differentiation, Rock2 positively regulates the maturation process 50. Overexpression of Pdlim5 significantly promotes C2C12 differentiation 51. Additionally, enhancement of Itgb1 activity improves muscle regeneration capacity in mice 52. However, despite these previous studies regarding the function of these proteins in the muscle, their isoform-specific effects on myogenic differentiation have not been well-studied, to the best of our knowledge. Consistent with our RNA-Seq data, we did not detect differential expression of the aforementioned genes (Figure S3E); however, their AS transition in UKO mice was validated. AS of Ppp3ca, Prkca, Rock2, Pdlim5 and Itgb1 were altered in response to loss of Rbm24 (Figure 6E). Therefore, aberrant muscle regeneration in Rbm24 KO mice may be partially attributed to the abnormal AS of these genes.

### Disruption of Rbm24 alters AS events that are essential for myogenesis and muscle regeneration

**Figure 7:**
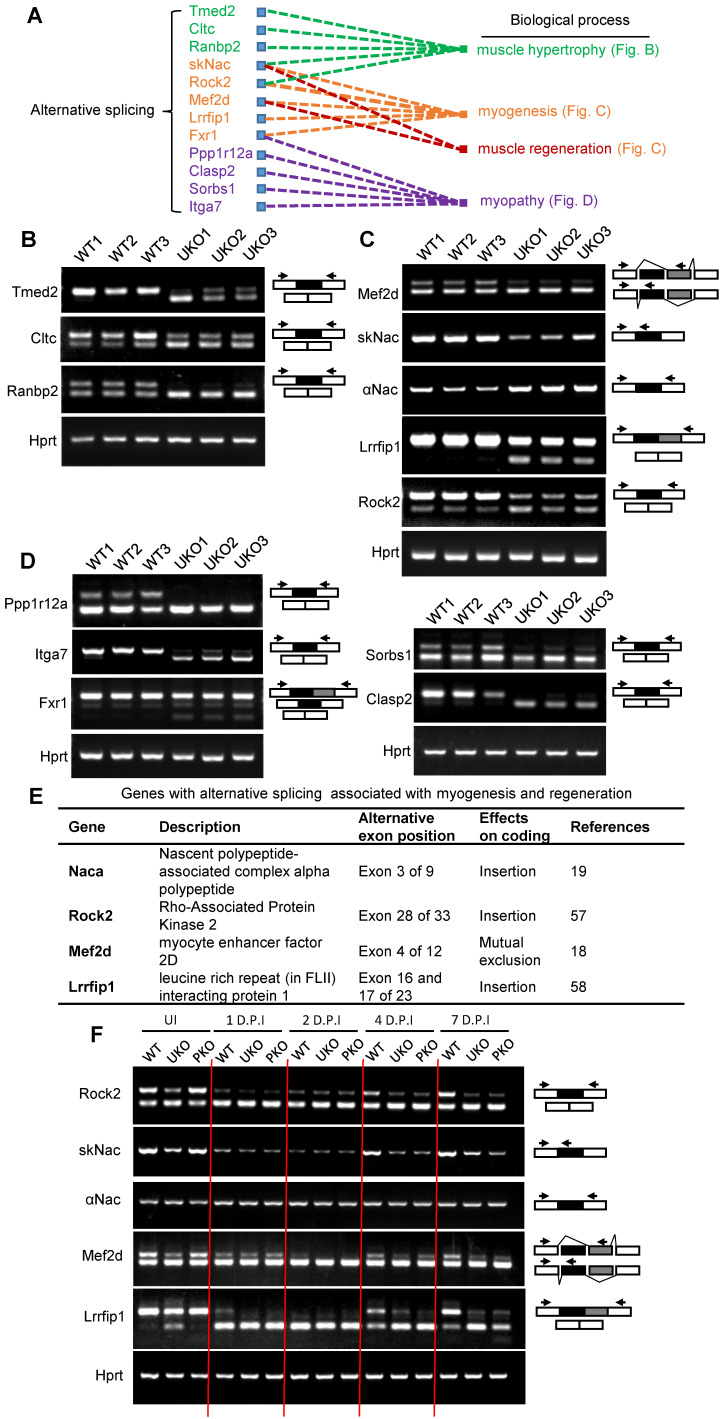
** Identify altered AS events in Rbm24 knockout mice which are indispensable for myogenesis and muscle regeneration.** (**A**) A network of AS events that are essential for muscle development and homeostasis. (**B-D**) RT-PCR analysis of AS events involved in (B) muscle hypertrophy, (C) myogenesis and muscle regeneration and (D) myopathy. The RNA samples were collected from TA muscle of 2-month-old mice 3 days after tamoxifen treatment. The arrowheads indicate the primer location and the dark or gray boxes represent the alternative exons. Hprt was used as the loading control. (**E**) A table showing the genes with isoform-specific functions in myogenesis or muscle regeneration. (**F**) RT-PCR analysis of AS of genes listed in (E) during regeneration. Naca is alternatively spliced to generate the skNac (muscle specific isoform) and αNac (ubiquitously expressed isoform). Total RNAs were extracted from injured TA muscle of 2-month-old mice at different time points following injury. The primers used for RT-PCR are indicated by the arrows. White box, flanking constitutive exons; gray and black box, alternative exons. Abbreviations: AS: alternative splicing; D.P.I: days post injury; RT-PCR: reverse transcription-polymerase chain reaction; TA muscle: tibialis anterior muscle.

We next determined which AS events were implicated in the regulation of myogenic differentiation. Simple GO analysis was unable to identify AS events that are involved in myogenic differentiation or muscle regeneration regulation. Therefore, we searched the AS events by manually retrieving literature. Relative literature was retrieved in Web of Science using the key words “gene symbol”, “alternative splicing” and “muscle”. For most of the AS events, there were no studies assessing the AS effects on muscle. However, we did find studies showing several Rbm24-regulated AS events, which were demonstrated to be involved in different biological processes, such as muscle hypertrophy, myogenesis, muscle regeneration and myopathy (Figure 7A). Briefly, simultaneous redirection of AS of Tmed2, Cltc, Snap23 and Trip10 in adult skeletal muscle leads to enlarged myofibers 53. The thickness of myotubes is altered after knockdown of the muscle specific isoform of Ranbp2 during C2C12 differentiation 54. The muscle specific isoform of Rock2 is required for myogenic hypertrophy in C2C12 50. Equally as important, Rbm24 also regulates AS of genes that are relevant to myopathy. Mis-splicing of Fxr1 was found in patients who suffered from facioscapulohumeral muscular dystrophy 55. Other genes, such as Itga7, Ppp1r12a, Pdlim5 and Clasp2, despite lack of detailed reports in skeletal muscle, have been shown to be abnormally spliced in muscular dystrophy 56. However, unfortunately, little is known regarding their isoform-specific function in the aforementioned diseases. We validated their splicing transitions in UKO mice (Figure 7B-D).

Importantly, we identified four genes Naca, Mef2d, Rock2, and Lrrfip1, the muscle-specific function of which have been reported to be essential for muscle regeneration or myogenic differentiation (Figure 7E). Briefly, overexpression of the mature isoform of Mef2d (Mef2dα2) significantly promotes muscle regeneration, whereas overexpression of Mef2dα1 does not 18. Conditional deletion of the muscle-specific isoform of Naca, skNac, significantly impairs muscle regeneration and hypertrophy in mice 19. It has also been shown that muscle-specific isoforms of Rock2 and Mef2d are essential for C2C12 differentiation 57. Furthermore, isoform-specific knockdown of Lrrfip1 significantly reduces myoblast fusion index during myoblast differentiation 58. Additionally, a comparison with the elaborate AS landscape during myoblast differentiation 57 also confirmed the AS transition of Mef2d, Naca, Rock2 and Lrrfip1 (Figure S4A and Table S4). We validated the AS transitions of these genes in Rbm24 knockout mice (Figure 7C).

We further analyzed the dynamic AS transition of Mef2d, Naca, Rock2 and Lrrfip1 during muscle regeneration in WT and Rbm24 knockout mice (Figure 7F). In the first 2 D.P.I, the mature isoforms of Rock2, Naca, Mef2d and Lrrfip1 decreased or even disappeared in WT and Rbm24 knockout mice due to myofiber degeneration. As regeneration occurred and increased, and nascent myofibers were generated, the disappeared mature isoforms reoccurred in the WT mice at 4 D.P.I, and the ratio of the mature isoforms increased as the nascent myofibers developed on 7 D.P.I. However, the expression of the mature isoforms did not notably increase in the Rbm24 knockout mice due to the lack of Rbm24 expression. These results indicate the involvement of AS of Rock2, Naca, Mef2d and Lrrfip1 in muscle regeneration and that loss of Rbm24 blocks their AS transition during regeneration.

### Rbm24 regulates AS to modulate myogenic differentiation

**Figure 8:**
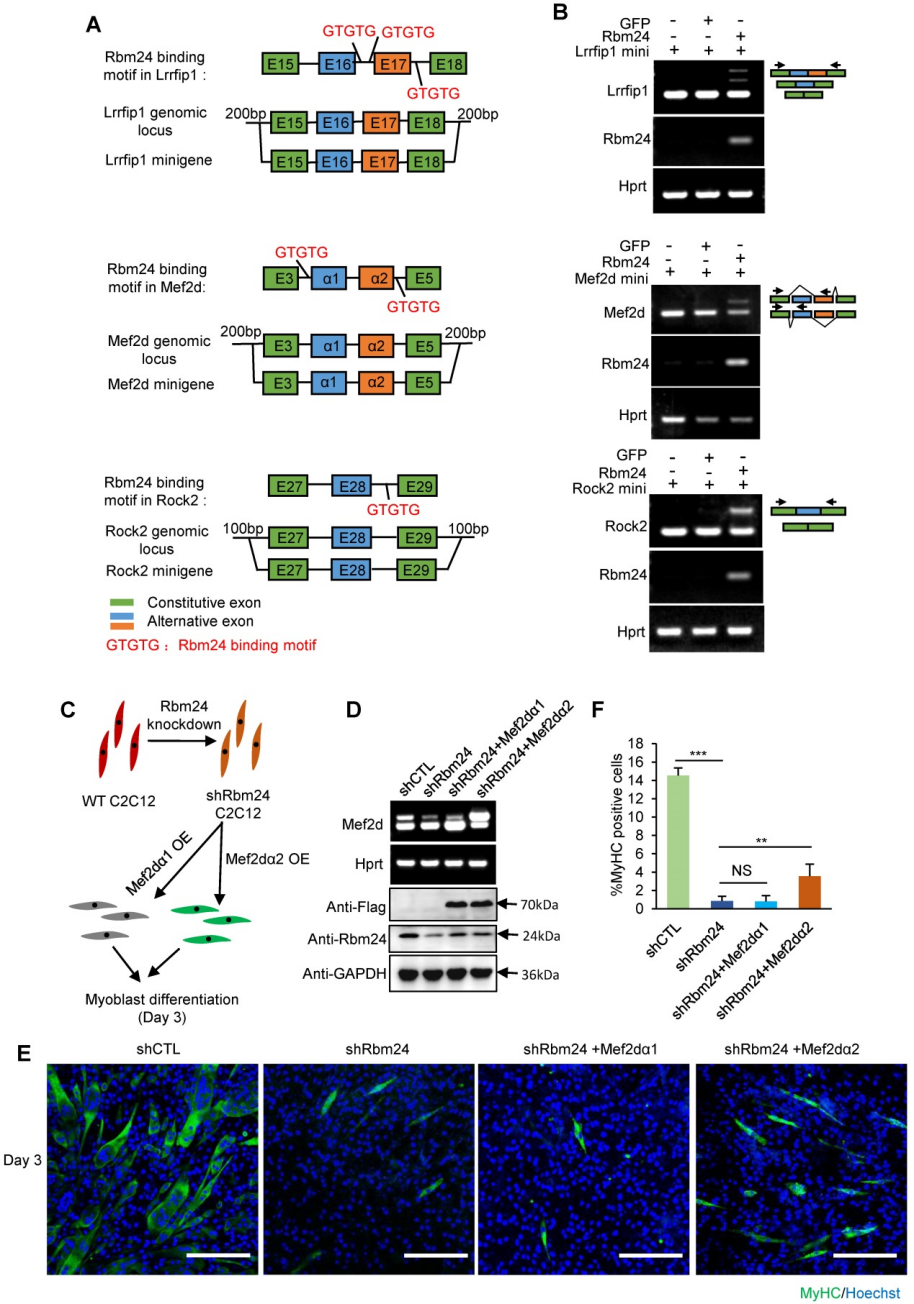
** Rbm24 regulates AS to modulate the myogenic process.** (**A**) Potential Rbm24 binding motif analyses and scheme of minigene construction of Mef2d, Rock2 and Lrrfip1. For Lrrfip1, potential Rbm24 binding sites were found 274 bp downstream of Exon 16, and 198 bp upstream and 125 bp downstream of Exon 17. For Mef2d, potential Rbm24 binding sites were found at 8 bp upstream of Exon α1 and 33 bp downstream of Exon α2. For Rock2, potential Rbm24 binding sites were found 120 bp downstream of Exon 28. (**B**) RT-PCR analysis of AS of minigene in response to Rbm24 overexpression. The GFP expression plasmid was used as a negative control. Green boxes, flanking constitutive exons; blue and orange boxes, alternative exons. The primers are indicated by arrowheads. (**C**) Diagram showing the scheme of construction of C2C12 cell lines in which Rbm24 expression was knocked down and different isoforms of Mef2d were overexpressed. (**D**) RT-PCR (Top panel) and western blot (Bottom panel) results showing successful overexpression of Mef2dα1 (undifferentiated isoform) and Mef2dα2 (differentiated isoform), and the knockdown of Rbm24 expression. Cells were treated with lentivirus expressing scrambled shRNA or Rbm24 shRNA (shRbm24), or lentivirus expressing either flag-tagged Mef2dα1 or Mef2dα2. Total RNAs and proteins were extracted from C2C12 3 days after switching to the differentiated medium. (**E**) Immunostaining analysis of MyHC-positive cells after 3 days of differentiation. Scale bars, 100 µm. (F) Statistical analysis of the MyHC-positive cells in (E). At least four different visual fields were analyzed to calculate the percentage of MyHC-positive cells in each group. Data are presented as mean ± SEM, **P < 0.01, unpaired *t*-test.

We observed the AS deficiency of Rock2, Naca, Mef2d and Lrrfip1 in Rbm24 knockout mice. Therefore, to further confirm the ability of Rbm24 to modulate AS of Mef2d, Rock2 and Lrrfip1, we investigated the role of Rbm24 in AS of Mef2d, Rock2 and Lrrfip1 in the 293T cell line (non-myogenic cell line) using the canonical minigene-splicing reporter system. AS of Naca has previously been shown to be directly regulated by Rbm24 20. Potential Rbm24 binding motif analyses and the scheme of minigene construction are shown in Figure 8A. According to the results, exogenous co-expression of Rbm24 with minigenes of either Mef2d, Rock2 or Lrrfip1 successfully recapitulated the AS pattern* in vivo*, but not in the control group (Figure 8B). These results confirm that Rbm24 is able to directly regulate the AS of Mef2d, Rock2 and Lrrfip1.

We further explored the effects of Rbm24-regulated AS events on myogenic differentiation in C2C12 cells. As the effect of AS of Mef2d on myogenic differentiation has previously been well-studied 18, 57, 59, 60 and Rbm24 knockdown has also been shown to disrupt the myogenic process during C2C12 differentiation 26, 61, we determined whether restoration of AS of Mef2d could rescue the differentiation defects in C2C12 cells in which Rbm24 expression was knocked down. We knocked down Rbm24 expression and overexpressed either the undifferentiated isoform of Mef2d (Mef2dα1) or the differentiated isoform of Mef2d (Mef2dα2) in C2C12 myoblasts by infecting C2C12 cells with the relevant lentiviruses. Differentiation of C2C12 cells was induced for 3 days (Figure 8C). Rbm24 expression was efficiently knocked down, and overexpression of different isoforms of Mef2d was also successful (Figure 8D, bottom panel). Rbm24-knockdown reduced the generation of Mef2dα2 (Figure 8D, top panel) and effectively blocked myogenic differentiation, as indicated by the notably decreased proportion of MyHC-positive cells (Figure 8E). More importantly, we observed a notable increase in the proportion of MyHC positive cells in the shRbm24-C2C12 cells that were overexpressing the differentiated isoform Mef2dα2, whereas this was not observed in the shRbm24-C2C12 cells that were overexpressing the undifferentiated isoform Mef2dα1 (Figure 8E-F). These results further demonstrate that Rbm24 can regulate myogenic differentiation by AS.

Together, these results show that Rbm24 regulated a series of AS events to modulate the myogenic differentiation process during skeletal muscle regeneration.

## Discussion

**Figure 9:**
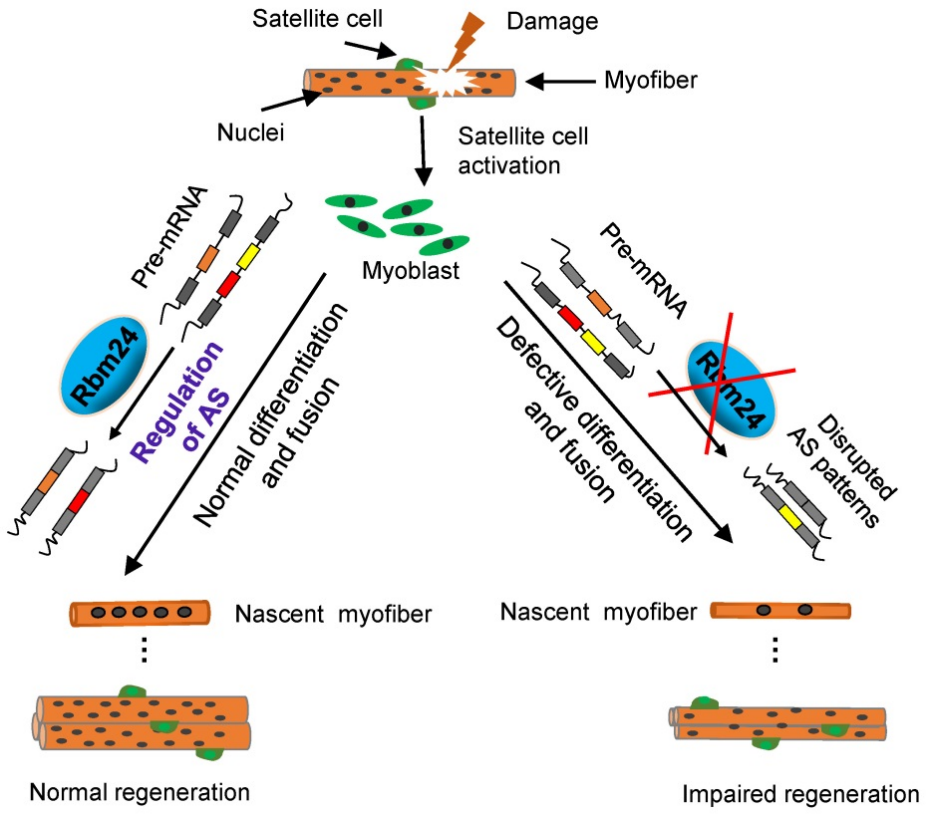
A working model shows that Rbm24 regulates AS to modulate muscle regeneration.

It has been demonstrated that Rbm24 serves a major role in development and pathological processes 20, 22, 24, 26. In the present study, we investigated the role of Rbm24 in adult skeletal muscle regeneration and found that Rbm24 positively regulated muscle regeneration, in part, by modulating myogenic fusion and differentiation through manipulation of AS. A proposed working model is presented in Figure 9.

We explored the role of Rbm24 in adult skeletal muscle using a conditional knockout strategy. Global knockout of Rbm24 led to a progressive loss of body weight and impaired muscle function as evidenced by decreased strength in the knockout mice (Figure S1). We observed abnormal muscle regeneration in the Rbm24 knockout mice 4 months after loss of Rbm24, and the aberrant regeneration was aggravated thereafter (Figure 1D). However, it was unclear whether Rbm24 deficiency directly caused these phenotypes, as the pathological consequences may also be due to malfunction of other organs, such as the heart 24, Notably, we also found that Rbm24 expression was elevated in a CTX-induced regeneration model and in mdx mice (Figure 2). Further regeneration analyses in Rbm24 knockout mice indicated that the loss of Rbm24 delayed the muscle regeneration process (Figure 3). Therefore, our results demonstrate that Rbm24 regulates adult skeletal muscle regeneration. Skeletal muscle regeneration has a characteristic hierarchical regulatory mechanism. Myoblast fusion is a crucial step for myogenesis and regeneration 5, 62. We found that Rbm24 controlled the myogenic fusion and differentiation process during muscle regeneration, as evidenced by the decreased MyHC^+^ myofibers and the reduced fusion index in the knockout group at 5 D.P.I. These results were further validated in the PKO mice, wherein Rbm24 was specifically knocked out in satellite cells. Consistent with our results, both mouse satellite cells and C2C12 cell differentiation experiments *in vitro* also indicated that Rbm24 is involved in the regulation of myogenic differentiation during myogenesis 27, 28. Thus, Rbm24 regulated muscle regeneration by modulating myogenic fusion and differentiation.

As a master splicing regulator, Rbm24 extensively regulates AS of genes involved in several biological processes, such as membrane organization, cytoskeleton organization, muscle contraction and ion homeostasis (Figure 6C). It should be noted that abnormal regeneration also occurred in the UKO mice 4 months after tamoxifen treatment even without CTX treatment (Figure 1D). Additionally, we did not observe altered Pax7 or Myod expression in the UKO mice 3 days after tamoxifen treatment (Figure S3A). Thus, abnormal regeneration was not the consequence of the direct activation of satellite cells due to deletion of Rbm24. It has been demonstrated that Rbfox1 regulates AS of muscle structure-related genes in adult skeletal muscle. Deletion of Rbfox1 alters muscle ultrastructure and increases sarcolemma fragility. As a result, Rbfox1 knockout mice are more susceptible to injury following exercise 63. In fact, Rbm24 knockout mice also exhibit muscle structure defects as indicated by the loss of the M-band in skeletal muscle 20. Furthermore, our results indicated decreased muscle strength in Rbm24 knockout mice (Figure S1D). Importantly, we also found that Rbm24 regulated AS of genes related to muscle structure, including in the sarcomere, myofibril and sarcolemma (Figure S3C). Therefore, the abnormal regeneration may be due to increased susceptibility to injury in Rbm24 knockout mice. Additional studies are required to address this issue.

Muscular dystrophy is usually accompanied by regeneration and dysregulation of AS 16. Several studies have attempted to clarify the contribution of AS to the pathogenesis of muscular dystrophy. For example, aberrant splicing of Bin1 was found in patients with congenital myotonic dystrophy, and this resulted in muscle weakness 64. However, few studies have focused on the effects of AS on muscle regeneration 17, 18. It is hypothesized that when the pathological muscle regeneration process is interrupted, the symptoms of muscular dystrophy may be aggravated. The present study provides evidence that Rbm24 deficiency results in dysregulation of AS and delayed muscle regeneration. Rbm24 regulated AS events that are implicated in several biological processes, including myogenesis, muscle regeneration and muscle hypertrophy (Figure 7A). In particular, we demonstrated that Rbm24 regulated AS of Mef2d, Naca, Rock2 and Lrrfip1, which were demonstrated to be essential for myogenesis and muscle regeneration.

Mef2d is mutually exclusively spliced to generate Mef2dα1 and Mef2dα2. *In vivo*, overexpression of Mef2dα2 promotes the regenerative process, whereas Mef2dα1 results in the opposite effect 18. skNac is the muscle specific-isoform of Naca that is generated by selective retention of exon 2. Knockout of skNac in mice severely disrupts the regenerative process 19. Similarly, we observed impaired muscle regeneration accompanied by decreased Mef2dα2 and skNac expression in Rbm24 knockout mice. Rock2 is a major effector of RhoA. Inclusion of the C-terminal exons of Rock2 impairs its kinase activity and facilitates myoblast fusion 57. Additionally, the long muscle-specific isoform of Lrrfip1 was also shown to be necessary for myogenesis. Isoform-specific knockdown of Lrrfip1 significantly reduces Myh3 and Myog expression, and impairs myoblast differentiation in C2C12 cells 58. Similarly, we observed the impaired myogenic differentiation capacity and the reduced expression levels of muscle-specific isoforms of Rock2 and Lrrfip1 in the Rbm24 knockout mice during regeneration. In the present study, we identified that the expression of the muscle-specific isoform of Mef2d in Rbm24-knockdown C2C12 cells partially restored the myogenic differentiation capacity. One recent study indicated that simultaneous redirecting AS of Tmed2, Cltc, Snap23 and Trip10 in adult skeletal muscle leads to enlarged myofibers in mice 53. Given the essential roles of the AS regulation of Mef2d, Naca, Rock2 and Lrrfip1 in myogenic process as mentioned above, it is worth exploring the combined effects of AS of these four factors on rescuing Rbm24-deletion caused defective myogenic differentiation in the future.

One attracting issue is whether Rbm24 plays a role in muscular dystrophy. A recent study reported the transcriptome alterations in patients with myotonic dystrophy 1 (DM1), which may assist in achieving a comprehensive understanding of the effects of AS on the pathogenesis of DM1 65. In that study, Rbm24 mRNA level was elevated in the TA muscle of DM1 patients based on its RNA-Seq data. However, it is not sure if any change occurred at Rbm24 protein level. Indeed, in the other study using immortalized human myotonic dystrophy muscle cell lines, Rbm24 was found to be abnormally spliced 66. It will be interesting to explore the possible correlation between the Rbm24 protein expression and muscular dystrophy and whether Rbm24 is dysfunctional in DM1 patients in the future.

Generally, AS is an outcome of the combined effects of several splicing factors. Despite the large number of splicing factors identified, little is known regarding the cooperative function of these splicing factors in controlling a biological process. It has been reported that Rbfox1 and Mbnl1 together control the splicing events altered in DM1 67. In the present study, we found that Rbm24 and Rbfox2 controlled a common set of AS events (Figure S4 and Table S5), including AS of Mef2d and Rock2, suggesting their potentially coordinated function in myogenic differentiation. Of note, we found that deletion of Rbm24 in skeletal muscle did impair AS of Mef2d without affecting Rbfox2 expression (Figure S4B), which may suggest that Rbfox2 cannot maintain sufficient generation of AS transition *in vivo* without Rbm24. Recently, it was demonstrated that knockout of both Rbfox1 and Rbfox2 in adult skeletal muscle results in a rapid loss of muscle mass and severely impairs muscle function 68. It will be interesting to explore the effects of simultaneous disruption of Rbm24 and Rbfox2 on muscle diseases.

In summary, our study highlights the key role of Rbm24-mediated AS in skeletal muscle regeneration. It will be of great interest to examine the effects of abnormal splicing events on muscle regeneration in patients with muscular dystrophy. Correction of the dysregulated splicing events, which are essential for muscle regeneration, may be a novel therapy for treating patients with muscular dystrophy.

## Supplementary Material

Supplementary figures and tables.

## Abbreviations

Rbm24: RNA binding motif protein 24
AS: alternative splicing
CTX: cardiotoxin
TA muscle: tibialis anterior muscle
mdx: X-linked muscular dystrophy
UI: uninjured
D.P.I: days post injury
CSA: cross-sectional areas
