# Supplementary material for: Rbm24 modulates adult skeletal muscle regeneration via regulation of alternative splicing
Source: Theranostics. 2020 Sep 11;10(24):11159–77. doi: 10.7150/thno.44389 (PMC7532667; doi:10.7150/thno.44389)
Supplement: Supplementary file 1 — Supplementary figures and tables. [file thnov10p11159s1.pdf]

Inventory of Supplementary Information

Table S1. Mouse tissue samples used for RNA-Seq, related to Figure 6, embedded in the Supplementary Materials document file.

Table S2. Differentially expressed genes identified by RNA-Seq in adult skeletal muscle, related to Figure 6, supplied as a separate spreadsheet file.

Table S3. Rbm24-regulated splicing events identified by RNA-Seq in adult skeletal muscle, related to Figure 6, supplied as a separate spreadsheet file.

Table S4. Rbm24-regulated alternative splicing events in skeletal muscle of mice which also occur during C2C12 differentiation, related to Figure 7, supplied as a separate spreadsheet file.

Table S5. Alternative splicing events in response to both Rbm24 and Rbfox2, embedded in the Supplementary Materials document file.

Table S6. Primer sequences for the alternative splicing assay, plasmid construction and various PCRs, supplied as a separate spreadsheet file.

Table S7. Annotation of the alternative exon loci in each gel image in Figures.

Supplementary Information

Supplemental Methods

Generation of the Rbm24 conditional knockout mice

The construction of conditional allele of Rbm24 has been described in detail in our previous study [1]. In brief, exon 2 and exon 3 of Rbm24 were flanked by loxP sites, which allowed for Cre recombinase-mediated knockout. We bred Rbm24$^{loxP/loxP}$ mice to the UBC-CreERT2 mice which contained a tamoxifen-inducible Cre recombinase-estrogen receptor fusion protein cassette driven by the UBC promoter for systemic Rbm24 knockout. Additionally, we bred Pax7-CreER mice with Rbm24$^{loxP/loxP}$ mice to generate satellite cell-specific Rbm24 knockout mice. The following genotype mice were used: UBC-CreERT2, Rbm24$^{loxP/loxP}$ (UKO) or Pax7-CreER, Rbm24$^{loxP/loxP}$ (PKO) and Rbm24$^{loxP/loxP}$ (WT). The sequences of genotyping primers are shown in Table S6. For inducible knockout of Rbm24, tamoxifen was administrated in 8-week-old male mice by intraperitoneal injection for 5 consecutive days. The knockout efficiency was validated both at the RNA and protein levels.

Cage Flip

Mice were placed on a grid for 60 s and allowed to acclimate to the environment, and then the cage was inverted for up to 3 min at a height of 50 cm. The time until the mice let go of the grid was recorded. Each mouse was tested three times with 5 min resting period between each test. The maximum hanging time of each mice was used for further analysis. Muscle strength is expressed in terms of physical impulse which is calculated as time (s) ∗ body mass (g) ∗ 0.00980665 N/g. A total of 9 mice per group were tested in triplicate. Data were analyzed using a Student’s t-test.

RNA-Seq and data analysis

RNA samples were extracted from hind limb TA muscle of 2-month-old WT or UKO male mice (n = 2) 3 days after tamoxifen administration using TRIzol reagent (Invitrogen). RNA quality was assessed by agarose gel electrophoresis and by evaluating the A260/A280 ratio. Reliable RNAs were pooled and sequenced using the

Illumina HiSeq system (Illumina). For each group, two biological replicates were used. Reads were aligned to mouse transcriptome (GRCm38/mm10) using Tophat [2]. Assembly of transcription was performed using Cufflinks. Gene expression was determined based on calculated fragments per kilobase of transcripts per million mapped reads (FPKM) and differentially expressed genes were identified by DESeq [3] with a false discovery rate (FDR) <5% and | log2 fold change | ≥1. Alternative splicing (AS) events were identified by Astalavista and rMATS with a FDR <5% and ΔIncLevelDifference ≥20% [4-6]. Gene ontology analysis was performed using DAVID.

RT-PCR and qRT-PCR

Total RNAs were prepared using TRIzol (Invitrogen) and cDNAs were synthesized using TransScript® One-Step gDNA Removal and cDNA Synthesis SuperMix (TransGen Biotech) according to the manufacturer's protocol. cDNA products were subjected to RT-PCR analysis. PCR products were separated on 1-3% agarose gel. qPCR was performed on an ABI 7500 (Applied Biosystems) using qPCR SYBR Green mix (Vazyme Biotech) according to manufacturer's protocol. Primers for various PCR are listed in Table S6.

Immunofluorescence staining

Muscle sections or cell slides were fixed in 4% paraformaldehyde at room temperature for 20 min and permeabilized for 20 min in 0.25% Triton X-100 in PBS. Then samples were blocked in 3.5% goat serum for 1 h at room temperature. Primary antibodies were incubated in blocking buffer at 4 ˚C overnight. The following day, the samples were washed by PBS and stained with the corresponding fluorescently labeled secondary antibodies (Alexa Fluor 488 or 555) for 1 h at room temperature. After washing with PBS, Hoechst (Sigma-Aldrich; 1:1,000) was used to stain the nuclei for 5 min. The primary antibodies used were: mouse anti-MyHC (DSHB; 1:250), rabbit anti-laminin (Sigma-Aldrich; 1:400), mouse anti-Pax7 (DSHB; 1:50), mouse anti-Myog (BD Biosciences; 1:200) and rabbit anti-Rbm24 (Abcam; 1:200).

Western blotting

TA muscles or cell pellets were washed with PBS and lysed in RIPA lysis buffer. Proteins were separated by SDS-PAGE gels and electrotransferred to a PVDF membrane (Pall Corporation). The PVDF membrane was blocked in 5% milk dissolved in TBST for 1 h at room temperature. Membranes were incubated with the primary antibodies at 4 °C overnight. Subsequently, the membrane was washed with TBST and incubated with the HRP-labeled secondary antibodies (goat anti-rabbit or anti-mouse) for 1 h at room temperature. After washing with TBST, signals were visualized by chemiluminescence. The following primary antibodies were used: Rabbit anti-Rbm24 (Abcam; 1:3,000), mouse anti-MyHC (DSHB; 1:500), mouse anti-Myogenin (BD Biosciences; 1:500), mouse anti-Pax7 (DSHB; 1:200), mouse anti-GAPDH (Transgene; 1:5,000) or mouse anti-Flag (Origene; 1:3,000).

Lentivirus production

We constructed the plasmid expressing shRNA directed against Rbm24 by inserting the custom oligos with *Xba*I and *Hpa*I restriction enzymes sites into the pLL3.7 vector that conferred G418 resistance. Two distinct Mef2d expression plasmids (Mef2dα1 and Mef2dα2) were constructed by inserting the amplified Mef2d fragments into the *Xba*I and *Mlu*I sites in the PLVCS-N-Flag vector that conferred puromycin resistance. These two plasmids were used for the rescue experiments. Lentivirus was produced by simultaneously transfecting the expression plasmid with the three package plasmids pVSVG, pMDL and pREV (2:1:1:1) into 293T cells using Lipofectamine® 3000 according to the manufacturer's protocol. After 48 h, the viral supernatant was collected and centrifuged at 500 * g for 5 min. The supernatant was further filtered using a 0.45 µm filter and then stored at -20 °C until use.

Supplementary Results

Progressive loss of muscle mass and strength in UKO mice

UKO exhibited smaller body sizes and body weight was significantly reduced 1 month after tamoxifen treatment (Figure S1A and B). Consistent with the decreased body weight, TA muscle weight was also significantly reduced in the UKO mice (Figure S1C). Furthermore, we determined whether muscle strength was also decreased as a result of the loss of muscle mass using an inverted cage flip test. The inverted cage flip test measured limb and pectoral strength by quantifying the time a mouse held on to the bottom of a cage [7]. In this experiment, 6 months old UKO mice were tested 3-4 months after tamoxifen administration. Based on the results of the cage flip test (Figure S1D), there was a significant loss of muscle strength observed in the UKO mice compared with the WT group.

Analysis of Rbm24 expression in mdx mice and satellite cells

We analyzed the expression of Rbm24 during mdx mice development. It has been demonstrated that mdx mice undergo pathological degeneration and regeneration waves after birth. In particular, the TA muscle of mdx mice show no signs of regeneration during the first two weeks after birth, whereas there is extensive regeneration seen in 4-week-old mdx mice, and the regenerative activity decreases thereafter [8, 9]. Interestingly, we found that Rbm24 expression was increased in TA muscle of 4-week-old mdx mice compared with the WT mice, but not in the 2-week-old mdx mice (Figures 2A-B and S2A-B). The increased expression levels of Rbm24 decreased as the mdx mice developed. Thus, Rbm24 expression mimics the regenerative activity in mdx mice, indicating the potential role of Rbm24 in regulation of muscle regeneration of mdx mice.

We further analyzed Rbm24 expression during satellite cell activation and differentiation according to the published RNA-Seq data [10]. As indicated in Figure S2C, Rbm24 expression was very low in quiescent satellite cells (WT freshly isolated satellite cells) and activated satellite cells (WT cultured satellite cells). Rbm24 was highly expressed in the differentiated Myog positive satellite cells (Figure S2D). The results of immunostaining also confirmed the results of RNA-Seq (Figure 2D).

Analysis of the RNA-Seq results

Based on our RNA-Seq data, we validated the differentially expressed genes in response to loss of Rbm24 (Figure S3A). In this part, Col12a1 and Pkg1 were assessed to determine differential expression. Consistent with the RNA-Seq results, both the expression levels of Col12a1 and Pkg1 were increased in the UKO mice. Interestingly, both the RNA-Seq results and qRT-PCR analyses showed that loss of Rbm24 did not alter Pax7 or Myod expression (Figure S3A), indicating that disruption of Rbm24 does not activate the satellite cells directly. We also examined the AS events identified in our RNA-Seq results. Both AS of Usp28 and Usp25 were analyzed as they have been reported to be alternatively spliced by Rbm24 in skeletal muscle [11]. The unknown AS events, such as AS of Cog1, Ubr3 and Tmem65 were also assessed (Figure S3B). All of the AS events tested showed notable transition in response to loss of Rbm24. These results suggest the RNA-Seq data are reliable and repeatable. Besides, GO analysis of alternatively spliced genes identified significant enrichment of genes relevant to muscle cell development (Figure 6C), muscle cell components (Figure S3C) and muscle cell functions (Figure S3D). We analyzed the expression of the genes enriched in cell differentiation or muscle cell development, including Ppp3ca, Prkca, Rock2, Pdlim5 and Itgb1. Consistent with the RNA-Seq results, their mRNA expression levels were not affected due to loss of Rbm24 (Figure S3E). In contrast, their abnormal splicing were identified following Rbm24 knockdown (Figure 6E).

We also compared our RNA-Seq results with the transcriptome data from other articles including the data from E11.5 heart [11] and Day 5 heart of Rbm24 knockout mice [1] (Figure S3F). We found only 20 genes were aberrantly spliced both in E11.5 heart and adult TA muscle of Rbm24 knockout mice. Besides, we identified 50 genes abnormally spliced both in Day 5 heart and adult TA muscle of Rbm24 knockout mice. Only 15 genes were abnormally spliced in E11.5 heart, Day 5 heart and TA muscle of Rbm24 knockout mice, including coro6, Naca, Fxr1, and Usp28 which have been well-studied in previous studies. Therefore, Rbm24 regulates AS in a context-dependent manner, and regulates different AS events at different developmental stages and in different tissues.

Rbm24 regulates a series of AS events involved in myogenic differentiation.

In order to identify additional Rbm24-regulated AS events which may play a role in myogenic differentiation, we compared the AS events in our RNA-Seq with the AS events occurring during C2C12 differentiation [12]. Excitingly, we found 62 Rbm24-regulated genes that were alternatively spliced during C2C12 differentiation, including Rock2, Naca, Lrrfip1 and Mef2d (Figure S4A and Table S4). Further analysis identified 47 Rbm24-regulated exons among the 62 genes were alternatively spliced during C2C12 differentiation, indicating their potential function in regulating myogenic differentiation. The genes identified are listed in Table S4.

Rbm24 and Rbfox2 regulate a common set of AS events

We noted that both Rbm24 and Rbfox2 regulated the AS of Mef2d and Rock2. Rbfox2 was also shown to regulate myogenesis by coordination of AS [12]. This prompts a potential cooperative mechanism in AS regulation between Rbm24 and Rbfox2 during the myogenic process. Thus, whether there were more AS events regulated by both Rbm24 and Rbfox2 was assessed. We compared genes which were alternatively spiced by Rbfox2 during myoblast differentiation with those alternatively spiced by Rbm24 in skeletal muscle. Interestingly, Rbm24 and Rbfox2 regulated AS of 28 common genes that are involved in myoblast differentiation (Figure S4A). Further analysis identified that 23 of the 28 genes were spliced at the same exon by both Rbm24 and Rbfox2 (Table S5). This further indicates the potential cooperative mechanism in AS regulation during the myogenic process between Rbm24 and Rbfox2.

Additionally, we assessed Rbfox2 expression in Rbm24 knockout mice. Rbm24 knockout in skeletal muscle did not alter the expression of Rbfox2 but notably decreased the muscle-specific exon of Mef2d, suggesting that Rbfox2 cannot maintain sufficient generation of muscle-specific isoform of Mef2d without Rbm24 (Figure S4B). These results hint that sufficient generation of AS transition *in vivo* may be dependent on the collaborative work of Rbfox2 and Rbm24.

References

1. Liu J, Kong X, Zhang M, Yang X, Xu X. RNA binding protein 24 deletion disrupts global alternative splicing and causes dilated cardiomyopathy. Protein Cell. 2019; 10: 405-16.
2. Trapnell C, Pachter L, Salzberg SL. TopHat: discovering splice junctions with RNA-Seq. Bioinformatics. 2009; 25: 1105-11.
3. Trapnell C, Roberts A, Goff L, Pertea G, Kim D, Kelley DR, et al. Differential gene and transcript expression analysis of RNA-seq experiments with TopHat and Cufflinks. Nat Protoc. 2012; 7: 562-78.
4. Foissac S, Sammeth M. ASTALAVISTA: dynamic and flexible analysis of alternative splicing events in custom gene datasets. Nucleic Acids Research. 2007; 35: W297-W9.
5. Shen S, Park JW, Lu ZX, Lin L, Henry MD, Wu YN, et al. rMATS: robust and flexible detection of differential alternative splicing from replicate RNA-Seq data. Proc Natl Acad Sci U S A. 2014; 111: E5593-601.
6. Liu J, Geng A, Wu X, Lin RJ, Lu Q. Alternative RNA Splicing Associated With Mammalian Neuronal Differentiation. Cereb Cortex. 2018; 28: 2810-6.
7. Chenette DM, Cadwallader AB, Antwine TL, Larkin LC, Wang J, Olwin BB, et al. Targeted mRNA Decay by RNA Binding Protein AUF1 Regulates Adult Muscle Stem Cell Fate, Promoting Skeletal Muscle Integrity. Cell Rep. 2016; 16: 1379-90.
8. Dangain J, Vrbova G. Muscle development in mdx mutant mice. Muscle Nerve. 1984; 7: 700-4.
9. Pastoret C, Sebille A. mdx mice show progressive weakness and muscle deterioration with age. J Neurol Sci. 1995; 129: 97-105.
10. Ryall JG, Dell'Orso S, Derfoul A, Juan A, Zare H, Feng X, et al. The NAD(+)-dependent SIRT1 deacetylase translates a metabolic switch into regulatory epigenetics in skeletal muscle stem cells. Cell Stem Cell. 2015; 16: 171-83.
11. Yang J, Hung LH, Licht T, Kostin S, Looso M, Khrameeva E, et al. RBM24 is a major regulator of muscle-specific alternative splicing. Dev Cell. 2014; 31: 87-99.
12. Singh RK, Xia Z, Bland CS, Kalsotra A, Scavuzzo MA, Curk T, et al. Rbfox2-coordinated alternative splicing of Mef2d and Rock2 controls myoblast fusion during myogenesis. Mol Cell. 2014; 55: 592-603.

Table S1. Mouse tissue samples used for RNA-Seq, related to Figure 6.

**Sample**	**Age**	**Sex**	**Total Reads**	**%Uniquely Mapped**
WT TA1	8 weeks	Male	39086524	84.02
WT TA2	8 weeks	Male	41945060	83.20
UKO TA1	8 weeks	Male	41432030	82.99
UKO TA2	8 weeks	Male	53925520	83.91

Legend: The 8-week-old male Rbm24$^{loxP/loxP}$ mice were used as the control and littermate UBC-CreERT2，Rbm24$^{loxP/loxP}$ mice as the KO mice. Each group had two biological replicates. Three days after tamoxifen treatment, TA muscle was dissected for RNA extraction and subjected to paired-end RNA-sequencing. The reads were mapped to the reference mouse genome (GRCm38/mm10) using Tophat.

Table S2. Differential expression genes identified by RNA-Seq in adult skeletal muscle.

gene symbol	baseMean	baseMeanA	baseMeanB	FoldChange	log2FoldChange	P-value	P-adj
Mmp13	486.2756308	73.60116451	898.9500972	12.21380264	3.610440533	1.5E-14	2.55E-10
Spp1	173.3708227	25.88663444	320.855011	12.39462054	3.631642199	2.29E-10	0.00000194
Col11a1	176.8542129	28.92102166	324.7874042	11.23014975	3.489305261	9.98E-10	0.00000472
Col12a1	575.4435126	157.0718388	993.8151864	6.327137915	2.661553043	1.21E-09	0.00000472
Ibsp	163.5583729	22.96452856	304.1522172	13.2444355	3.727314449	1.39E-09	0.00000472
Klk1b22	28.77655142	0	57.55310283	Inf	Inf	5.15E-09	0.0000146
Tnn	203.9218239	45.08816493	362.7554829	8.045470103	3.008176721	6.63E-08	0.000160648
Postn	703.9582696	244.4760813	1163.440458	4.758913231	2.25063215	9.55E-08	0.000202554
Adamts8	252.9407933	90.50980782	415.3717788	4.589246059	2.198257161	0.000000201	0.000378203
Cilp	228.6951037	93.36932843	364.020879	3.898720116	1.963000589	0.00000172	0.002649448
Col1a1	5545.507088	2022.154136	9068.86004	4.484752115	2.165028245	0.00000282	0.003713998
Ogdhl	50.58238592	13.50733729	87.65743454	6.489616173	2.698133153	0.00000392	0.004751785
Fmod	269.5813667	111.5799482	427.5827853	3.832075497	1.938125985	0.000009	0.010175296
Tnc	131.9264133	37.54161713	226.3112095	6.028275465	2.591745343	0.0000131	0.013095311
AI662270	41.39613253	6.16050414	76.63176091	12.43920289	3.636822135	0.0000249	0.021081004
Col16a1	232.6844103	90.52606307	374.8427575	4.140716439	2.049880409	0.0000333	0.025648597
Krt14	230.2056824	363.3326149	97.07874985	0.267189748	-1.904063441	1.14E-06	0.00193064
Avil	169.9068181	280.2236327	59.59000344	0.212651599	-2.233436395	2.85E-06	0.003713998
Mt2	174.93366	282.6288012	67.23851872	0.237903987	-2.071548643	1.13E-05	0.01196442
BC037156	110.4264159	177.6474541	43.20537768	0.243208539	-2.039734215	1.47E-05	0.013892262
Erdr1	711.5305107	1083.66491	339.3961117	0.313192859	-1.674876774	2.06E-05	0.018411982
Krt5	233.6689696	351.5901989	115.7477402	0.329212079	-1.602910826	2.69E-05	0.021691378
Myh8	773.5396146	345.9829724	1201.096257	3.471547309	1.795578832	2.02E-06	0.017450566

baseMean	normalized counts, averaged over all samples
baseMeanA	normalized counts from WT mice
baseMeanB	normalized counts from Rbm24 knockout mice
P-adj	adjusted P-value

Table S3. Rbm24-regulated alternative splicing events identified by RNA-Seq in adult skeletal muscle.

SE

gene symbol	chr	exonStart	exonEnd	upstreamES	upstreamEE	downstreamES	downstreamEE	P-value	IncLevelDiffer
Lrrfip1	chr1(+)	91082140	91082179	91079030	91079081	91084945	91085005	0	0.817
Ppfibp2	chr7(+)	107705283	107705466	107697566	107697698	107708779	107708872	1.04E-13	0.73
Atp11a	chr8(+)	12863761	12863862	12861639	12861724	12864919	12868728	1.17E-09	0.704
Clasp2	chr9(+)	113860119	113860137	113854172	113854346	113862185	113862287	3.85E-10	0.635
Ppfibp1	chr6(+)	146996808	146996901	146996389	146996482	146998166	146998281	0	0.629
Pex11a	chr7(-)	79740594	79740640	79735956	79737910	79742909	79743080	5.78E-08	0.608
R3hdm1	chr1(+)	128162768	128162858	128153188	128153399	128167892	128168007	8.76E-11	0.586
Slc8a3	chr12(-)	81216808	81216827	81214065	81214169	81314259	81316104	5.31E-05	0.574
Cad	chr5(+)	31075470	31075521	31075088	31075185	31075808	31075853	1.11E-06	0.573
Coro6	chr11(+)	77466765	77466975	77466505	77466599	77468265	77468370	0	0.553
Ash2l	chr8(-)	25830374	25830392	25828765	25828859	25831272	25831348	2.21E-10	0.552
Uso1	chr5(+)	92183481	92183502	92181897	92182109	92187234	92187341	7.77E-16	0.535
Ubr3	chr2(+)	69971401	69971434	69971151	69971233	69973148	69973609	0	0.528
Kif2a	chr13(-)	106969703	106969817	106968637	106968788	106974494	106974562	0.000165784	0.498
Ppp1r12a	chr10(+)	108248955	108249069	108243005	108243130	108249434	108249650	0	0.485
Sorbs1	chr19(-)	40361041	40361104	40352517	40352565	40363201	40363265	3.12E-08	0.479
Arl3	chr19(-)	46541442	46541510	46531108	46531386	46542351	46542537	0	0.471
Cog1	chr11(+)	113661658	113661693	113661139	113661218	113662206	113662400	1.56E-09	0.467
Abcd3	chr3(-)	121787979	121788049	121786008	121786071	121791733	121791822	2.22E-16	0.444
Lrrfip2	chr9(+)	111179176	111179221	111179046	111179097	111179872	111179920	0	0.441
Pip5k1a	chr3(-)	95089148	95089187	95082790	95082828	95106375	95106856	1.15E-05	0.435
Clip1	chr5(-)	123635173	123635278	123630452	123631169	123640493	123640554	0.000115708	0.425
Clasp2	chr9(+)	113858580	113858604	113854172	113854346	113862185	113862287	2.45E-06	0.423
Fbxw11	chr11(+)	32652747	32652904	32642903	32642935	32711771	32711997	2.73E-05	0.422
Ranbp2	chr10(+)	58481318	58481396	58476156	58480819	58483028	58483199	4.23E-06	0.42
Clasp2	chr9(+)	113862435	113862462	113862185	113862287	113868547	113868718	1.00E-10	0.42
Sorbs1	chr19(-)	40361428	40361503	40352517	40352565	40363201	40363265	7.71E-09	0.416
Prkca	chr11(-)	107945556	107945604	107933386	107939680	107951465	107951606	2.24E-12	0.412
Txnl1	chr18(-)	63665651	63665694	63662800	63664332	63671581	63671686	0	0.411
Tbc1d13	chr2(+)	30135408	30135552	30134796	30134870	30137120	30137182	0.000204396	0.411
Txnl1	chr18(-)	63665737	63665915	63662800	63664332	63671581	63671686	0	0.398
Rock2	chr12(+)	16973439	16973610	16972980	16973149	16974841	16974912	4.81E-10	0.389
Neb	chr2(-)	52161370	52161498	52160655	52160748	52162067	52162172	0	0.387
Tpd52	chr3(-)	8936438	8936480	8934991	8935040	8943550	8943577	7.71E-06	0.384
Fry	chr5(+)	150461388	150461397	150457744	150457915	150466243	150466441	2.20E-08	0.379
Kif13a	chr13(-)	46760262	46760304	46752080	46752872	46761272	46761424	7.43E-09	0.378
Tmem65	chr15(-)	58807077	58807158	58797200	58797245	58822842	58823530	1.88E-12	0.373
Ywhae	chr11(+)	75762697	75762742	75759291	75759428	75764619	75764623	0	0.37
Tmed2	chr5(+)	124543486	124543507	124543079	124543164	124546895	124547003	0	0.368
Naca	chr10(+)	128039170	128045086	128036488	128036560	128046167	128046253	0	0.365

Dysf	chr6(+)	84096476	84096518	84087791	84087874	84097707	84097823	5.55E-06	0.365
Rnf115	chr3(+)	96755152	96755204	96754334	96754393	96757976	96758034	2.61E-06	0.362
Lrrfip1	chr1(+)	91087058	91087124	91084945	91085005	91091601	91091667	2.08E-06	0.361
Sorbs1	chr19(-)	40361041	40361104	40352517	40352565	40361428	40361503	8.92E-05	0.359
Atg13	chr2(-)	91682802	91682901	91682025	91682136	91684668	91684759	0.000143669	0.359
R3hdm2	chr10(+)	127465130	127465184	127464981	127465013	127471767	127472000	0	0.354
Mpzl1	chr1(-)	165601749	165601855	165592239	165593677	165604609	165604742	4.54E-06	0.354
Pfkfb2	chr1(-)	130697016	130698085	130689182	130691148	130698678	130698743	6.00E-06	0.353
Ank1	chr8(+)	23132096	23132471	23123831	23123978	23132561	23132634	3.60E-05	0.351
Mapt	chr11(+)	104305312	104305409	104302360	104302410	104306625	104306734	0.000774744	0.35
Gtf2a1	chr12(-)	91575583	91575791	91572565	91572633	91586699	91586801	6.64E-07	0.349
Phkb	chr8(+)	86025197	86025288	86024280	86024338	86026456	86026547	4.91E-07	0.348
Snx6	chr12(-)	54768711	54768766	54768050	54768142	54770718	54770982	1.11E-05	0.348
Coro6	chr11(+)	77467749	77467869	77466505	77466599	77468265	77468413	0	0.347
Abcc1	chr16(+)	14362178	14362294	14361557	14361643	14389806	14389983	1.62E-09	0.343
Ank1	chr8(+)	23132096	23132483	23123831	23123978	23132561	23132634	8.23E-05	0.341
Mia3	chr1(-)	183329585	183329657	183328203	183328345	183329745	183329819	0	0.339
Clasp1	chr1(+)	118541674	118541698	118539265	118539369	118543101	118543346	0.000159588	0.338
Rilpl1	chr5(-)	124495898	124495917	124493651	124493844	124501839	124501932	0.000200332	0.33
Neb	chr2(-)	52151282	52151456	52149589	52149697	52159476	52159569	0	0.329
Srsf9	chr5(+)	115328346	115328430	115327394	115327499	115330497	115330658	1.46E-12	0.329
Ppp3cb	chr14(-)	20511473	20511500	20509415	20509500	20515522	20515600	8.38E-12	0.329
Neb	chr2(-)	52157831	52157924	52149589	52149697	52159476	52159569	0	0.323
Rhot1	chr11(+)	80264243	80264450	80257513	80257636	80265741	80267486	0.000120412	0.321
Ppp3ca	chr3(+)	136932010	136932040	136928536	136928619	136935028	136935779	0	0.319
Neb	chr2(-)	52156354	52156447	52150536	52150629	52157831	52157924	6.11E-05	0.317
Pex11a	chr7(-)	79740164	79740280	79735956	79737910	79742909	79743080	3.54E-08	0.316
Arl2bp	chr8(+)	94667029	94667230	94666723	94666901	94667596	94667658	2.49E-10	0.315
Las1l	chrX(-)	95947397	95947448	95946866	95947073	95947769	95947855	1.20E-07	0.315
Mlf1	chr3(+)	67391009	67391022	67384577	67384698	67392891	67392975	7.94E-07	0.314
Ssr1	chr13(-)	37983094	37983109	37979742	37979806	37983392	37983486	1.01E-06	0.31
Pkm	chr9(+)	59663039	59663156	59661414	59661471	59665199	59665366	0.000340707	0.308
Srsf9	chr5(+)	115331449	115331539	115330497	115330658	115332116	115332289	1.39E-06	0.306
Car14	chr3(-)	95901458	95901480	95901092	95901272	95902110	95902131	5.71E-05	0.303
Obscn	chr11(-)	59054773	59055037	59054161	59054425	59067065	59067329	4.44E-16	0.302
Stim2	chr5(+)	54110115	54110139	54109702	54109870	54110536	54110637	1.25E-05	0.301
Mlip	chr9(-)	77216976	77217048	77190671	77190737	77239391	77239784	0.001121544	0.299
Fermt2	chr14(-)	45463245	45463269	45462714	45462735	45464723	45464945	4.06E-13	0.299
Csde1	chr3(+)	103036574	103036721	103028788	103029212	103038691	103038890	1.85E-08	0.295
Lpin1	chr12(-)	16571163	16571262	16568389	16568519	16573659	16573785	3.61E-14	0.293
Arfgap2	chr2(+)	91269069	91269111	91268451	91268508	91269451	91269504	1.73E-06	0.293
Vps39	chr2(-)	120346856	120346889	120346327	120346392	120350163	120350229	2.57E-05	0.293
Meaf6	chr4(+)	125103429	125103570	125102838	125103031	125107651	125107709	0.000483733	0.292
Reep1	chr6(+)	71805342	71805494	71795146	71795324	71807790	71810246	1.10E-13	0.291

Cltc	chr11(-)	86696175	86696213	86695316	86695690	86697264	86697340	4.62E-07	0.291
Mpp5	chr12(+)	78828617	78828774	78826260	78826332	78829652	78829820	0.000103887	0.29
Tpm3	chr3(+)	90091012	90091091	90090009	90090079	90099527	90100896	4.16E-12	0.288
Prpf18	chr2(-)	4648096	4648123	4645568	4645673	4648240	4648318	1.22E-07	0.287
Adss	chr1(-)	177770439	177770512	177769924	177770022	177771140	177771265	2.03E-05	0.286
Rmnd1	chr10(-)	4428368	4428465	4427522	4427691	4429076	4429165	5.02E-12	0.285
Usp25	chr16(+)	77097648	77097762	77093602	77093742	77107884	77108063	7.32E-05	0.28
Neb	chr2(-)	52151363	52151456	52150536	52150629	52159476	52159569	2.04E-09	0.279
Kif13a	chr13(-)	46808156	46808276	46802515	46802729	46808890	46809057	2.58E-05	0.279
Ccdc85c	chr12(-)	108216786	108216825	108211514	108211622	108221708	108221782	7.48E-08	0.276
Nudt6	chr3(-)	37415650	37415692	37412359	37412415	37416700	37416904	0.000930924	0.275
Trdn	chr10(+)	33333231	33333248	33332435	33332465	33334981	33335014	6.43E-10	0.273
Arhgap12	chr18(-)	6111684	6112439	6069830	6070091	6115813	6115850	9.62E-05	0.271
Obscn	chr11(-)	59054773	59055037	59052444	59052711	59067065	59067329	4.41E-07	0.27
Mapt	chr11(+)	104307880	104307934	104306625	104306758	104310268	104310534	1.29E-05	0.269
Zfp746	chr6(-)	48082153	48082267	48067168	48067360	48083092	48083174	1.53E-05	0.267
R3hdm2	chr10(+)	127481692	127481794	127476519	127476698	127483975	127484218	1.38E-05	0.266
Rnps1	chr17(+)	24418037	24418228	24415040	24415162	24418456	24418612	0.000382901	0.266
Mybpc1	chr10(-)	88521276	88521335	88519933	88519943	88522966	88523187	0	0.264
Cyld	chr8(+)	88718289	88718298	88709890	88709996	88719297	88719396	2.91E-06	0.261
Mef2d	chr3(+)	88161780	88161951	88161190	88161381	88162957	88163087	3.67E-06	0.261
Chtop	chr3(-)	90505394	90505478	90503095	90503282	90506107	90506261	6.70E-06	0.26
Qrich1	chr9(+)	108519717	108519753	108518766	108518826	108528588	108528859	0.00119629	0.26
Neb	chr2(-)	52150536	52150629	52149589	52149697	52159476	52159569	0	0.256
Fxr1	chr3(+)	34064973	34065054	34064212	34064319	34068916	34069342	0	0.256
Rock2	chr12(+)	16982650	16982747	16977888	16978102	16984637	16987823	1.37E-07	0.256
Psma4	chr9(+)	54951343	54951428	54950814	54950903	54952081	54952108	1.82E-09	0.256
Fxr1	chr3(+)	34068160	34068252	34064118	34064319	34068916	34069345	0	0.255
Lims1	chr10(+)	58418647	58418835	58418398	58418474	58421453	58421664	0.000202918	0.254
Taf12	chr4(+)	132281993	132282078	132274415	132274487	132282793	132283045	0.001067477	0.254
Mybpc1	chr10(-)	88521276	88521335	88518338	88518569	88522966	88523187	0	0.253
R3hdm2	chr10(+)	127481692	127481755	127476519	127476698	127483975	127484218	2.54E-06	0.252
Calu	chr6(+)	29361559	29361753	29361293	29361487	29366039	29366206	1.09E-07	0.252
Coro6	chr11(+)	77467278	77467343	77466505	77466599	77468265	77468413	0.001408138	0.251
Itgb1	chr8(+)	128726982	128727063	128726019	128726186	128732022	128732389	9.86E-14	0.25
Tead1	chr7(+)	112842039	112842102	112839415	112839480	112856797	112856932	1.66E-05	0.25
Itga7	chr10(+)	128956610	128956723	128953945	128954071	128957620	128958279	1.40E-11	0.249
Msl3	chrX(-)	168672262	168672345	168671794	168671890	168673727	168673898	6.79E-05	0.249
Hnrnph2	chrX(+)	134602917	134602962	134602699	134602762	134604854	134607054	9.59E-05	0.247
Fam168b	chr1(-)	34824019	34824055	34819866	34820009	34828766	34828847	2.24E-05	0.246
Ndrg3	chr2(-)	156929931	156929970	156927344	156928881	156931215	156931267	1.04E-08	0.245
Neb	chr2(-)	52153055	52153148	52150536	52150629	52157831	52157924	7.52E-07	0.242
Neb	chr2(-)	52151363	52151456	52149589	52149697	52159476	52159569	0	0.241
Palld	chr8(-)	61514987	61515034	61511432	61513524	61515202	61515336	0.000288005	0.238

Neb	chr2(-)	52157290	52157383	52150536	52150629	52157831	52157912	4.71E-07	0.237
Lsmem1	chr12(-)	40185284	40185420	40175492	40177228	40199206	40199490	0.000821033	0.237
Ppp3cb	chr14(-)	20511473	20511500	20509415	20509497	20515522	20515600	4.37E-05	0.235
Hgsnat	chr8(-)	25973580	25973690	25972841	25972957	25976524	25976753	0.000378235	0.235
Lrrfip1	chr1(+)	91088633	91088711	91084945	91085005	91091601	91091667	9.85E-07	0.234
Obscn	chr11(-)	59064186	59064450	59054161	59054425	59067065	59067329	1.32E-07	0.231
Cobl	chr11(-)	12373328	12373373	12369593	12369691	12375809	12376038	0	0.228
Ndufaf5	chr2(+)	140202854	140203036	140193582	140193643	140203287	140203370	0.00020981	0.227
Mybpc1	chr10(-)	88575128	88575164	88573415	88573529	88585659	88585695	0	0.226
Akap9	chr5(+)	4031110	4031149	4029839	4030052	4032680	4032913	1.57E-05	0.225
Tacc2	chr7(+)	130744526	130744616	130742129	130742301	130751189	130751333	2.73E-14	0.225
Ankrd33b	chr15(-)	31325089	31325215	31304991	31305132	31367038	31367726	3.92E-05	0.224
Usp28	chr9(+)	49032508	49032688	49032213	49032353	49033576	49033672	1.95E-12	0.222
Mlip	chr9(-)	77229484	77230954	77190671	77190737	77239391	77239784	0.000128413	0.222
Ppp1r12a	chr10(+)	108233135	108233175	108230436	108230596	108233998	108234143	1.67E-06	0.219
Rilpl1	chr5(-)	124496173	124496270	124493651	124493844	124501839	124501952	0.000405519	0.219
Reep1	chr6(+)	71805330	71805494	71799165	71799201	71807790	71810246	2.15E-11	0.217
Kcnma1	chr14(-)	23336039	23336120	23330839	23330970	23336944	23337058	9.47E-06	0.217
Slc38a10	chr11(-)	120108466	120108553	120106433	120106610	120109431	120109536	0.000191387	0.216
Exoc3	chr13(-)	74207545	74207591	74206906	74207097	74208657	74208709	1.52E-06	0.215
Cd47	chr16(+)	49899855	49899900	49898039	49898132	49906780	49906812	0.000758076	0.214
Acot8	chr2(-)	164802996	164803133	164799557	164799945	164804689	164804822	0.000839648	0.213
Cltc	chr11(-)	86701060	86701081	86697264	86697340	86702208	86702430	0.000159345	0.211
Ubap2l	chr3(-)	90031295	90031370	90028324	90028377	90034064	90034177	3.50E-06	0.21
Ablim2	chr5(+)	35831685	35831742	35828123	35828182	35833052	35833130	3.74E-13	0.21
Sema6c	chr3(+)	95171790	95171886	95171557	95171602	95172254	95174024	3.25E-05	0.21
Nudt13	chr14(+)	20295087	20295266	20294722	20294792	20300586	20300679	0.000111466	0.21
Phtf2	chr5(-)	20812940	20812993	20807403	20807473	20813239	20813296	6.22E-05	0.208
Fbxw11	chr11(+)	32652747	32652904	32642903	32642935	32680200	32680302	0.000451942	0.206
Nudt13	chr14(+)	20295087	20295268	20294745	20294792	20300586	20300679	0.000535889	0.206
Diaph1	chr18(-)	37847945	37848004	37843602	37845498	37851550	37851637	1.52E-10	0.204
Mff	chr1(+)	82741817	82741976	82735418	82735507	82747082	82747142	3.22E-05	0.203
Bnip3l	chr14(-)	66988885	66988941	66985238	66987775	66989134	66989284	0.000246869	0.203
Pdlim3	chr8(+)	45908468	45908656	45900479	45900559	45915128	45915259	0.0003718	0.2
Lrrfip1	chr1(+)	91108579	91108672	91107298	91107390	91112156	91112297	0.0000262	0.185
Ccnl2	chr4(+)	155819705	155819932	155818586	155818664	155820328	155820428	0.000148076	-0.201
Ric8b	chr10(+)	84970728	84970824	84969862	84970091	84980047	84980192	0.000603069	-0.201
Pigq	chr17(-)	25931673	25931827	25931461	25931573	25932104	25932231	0.000130055	-0.203
Spopl	chr(-)	23545443	23545579	23544745	23544897	23571842	23572104	0.000107668	-0.204
Mafg	chr11(-)	120630487	120630647	120629817	120629881	120631242	120631440	0.000805968	-0.209
Upf3b	chrX(-)	37099065	37099104	37096857	37097018	37099506	37099689	1.29E-06	-0.214
Camk2b	chr11(-)	5976764	5976893	5972812	5972888	5979672	5979721	0.001093124	-0.216
Wdyhv1	chr15(+)	58150981	58151062	58150605	58150656	58152596	58152745	9.37E-08	-0.222
Celf2	chr2(-)	6549831	6549975	6546239	6547213	6553784	6553982	0.00047644	-0.223

Ifi27	chr12(+)	103437452	103437524	103436562	103436682	103437675	103437705	0.001112669	-0.229
Tnnt3	chr7(+)	142514158	142514366	142513532	142513705	142515775	142516008	7.28E-06	-0.232
Gpbp1	chr13(-)	111447895	111447955	111440631	111440816	111448932	111448999	9.22E-06	-0.234
Ank3	chr10(+)	70006802	70006853	70004609	70004991	70008777	70008864	0.001020413	-0.234
Rreb1	chr13(+)	37941542	37941704	37929557	37932489	37946856	37947663	0.000484832	-0.236
Pum1	chr4(+)	130753941	130754178	130752575	130752872	130762753	130763021	0.000943248	-0.24
Hcfc2	chr10(+)	82709008	82709217	82702442	82702603	82709963	82710048	0.000635687	-0.244
Ryr1	chr7(-)	29044918	29044936	29043848	29043929	29045603	29045677	0	-0.251
Slc22a23	chr13(-)	34193248	34193272	34192155	34192279	34193393	34193426	7.73E-07	-0.253
Phkb	chr8(+)	86026456	86026547	86025197	86025288	86029481	86029684	7.49E-07	-0.261
Huwe1	chrX(+)	151859454	151859688	151857807	151857913	151860166	151860546	0.001113899	-0.263
Ythdc1	chr5(+)	86809304	86809340	86804495	86804591	86815628	86815730	0.000448125	-0.274
Zfp62	chr11(+)	49214554	49214654	49213589	49213642	49215063	49218816	0.00063644	-0.279
Hnrnpr	chr4(+)	136314089	136314255	136311684	136311747	136316360	136316479	3.93E-06	-0.28
Ank3	chr10(+)	70006799	70006853	70004609	70004991	70008777	70008864	0.000232871	-0.281
Papola	chr12(+)	105811036	105811109	105809523	105809662	105812273	105812394	0.000206811	-0.282
Klhl26	chr8(-)	70455465	70455648	70450679	70453019	70476849	70476950	0.000536474	-0.283
Hnrnpr	chr4(+)	136314086	136314255	136311590	136311747	136316360	136316479	3.11E-06	-0.286
Tatdn1	chr15(-)	58909576	58909649	58905740	58905811	58916151	58916192	5.91E-06	-0.286
Smad2	chr18(+)	76302078	76302216	76299782	76299995	76302382	76302642	0.00014589	-0.29
Per2	chr1(-)	91423689	91424486	91421367	91421554	91427738	91427978	0.000175668	-0.322
Socs6	chr18(-)	88886991	88887052	88870249	88870918	88887208	88887247	0.00029919	-0.323
Babam2	chr5(+)	31701825	31701927	31698049	31698077	31702030	31702092	3.85E-08	-0.352
Snhg5	chr9(-)	88521917	88521975	88521649	88521716	88522306	88522381	0.000706106	-0.361
Rsad1	chr11(-)	94548158	94548363	94544419	94544785	94548470	94548604	0.000341226	-0.366
Kansl1l	chr1(-)	66802105	66802168	66777966	66778108	66817373	66817562	0.0001558	-0.377
Mettl3	chr14(-)	52298639	52298815	52298034	52298085	52299700	52300105	2.00E-05	-0.385
Fam193a	chr5(+)	34440347	34440590	34440120	34440233	34443300	34443454	2.28E-06	-0.41
Tmem234	chr4(+)	129601869	129601961	129601406	129601533	129602194	129602261	5.96E-05	-0.421
Ctbs	chr3(+)	146454162	146454485	146450477	146450630	146454947	146455156	0.0007709	-0.46
Clasp1	chr1(+)	118547655	118547658	118545392	118545456	118547864	118548028	1.09E-06	-0.469
Dbt	chr3(+)	116520236	116520360	116513127	116513191	116533254	116533436	2.86E-08	-0.535
Abcd3	chr3(-)	121786001	121786148	121784472	121784570	121787979	121788049	2.54E-10	-0.57

A3SS

gene	chr	longExonStart	longExonEnd	shortES	shortEE	flankingES	flankingEE	P-Value	IncLevelDiffer
Pdlim5	chr3(-)	142312122	142312541	142312122	142312214	142314405	142314448	7.86616E-06	0.637
Ank1	chr8(+)	23132096	23132634	23132561	23132634	23123831	23123978	7.28723E-05	0.331
Tnnt3	chr7(+)	142514155	142514366	142514325	142514366	142513532	142513705	2.21156E-13	-0.296
Got2	chr8(-)	95865056	95865268	95865056	95865247	95866820	95867207	0.000142511	0.253
Odf2l	chr3(+)	145145875	145148616	145148530	145148616	145144311	145144416	2.98E-07	0.242
Lamp1	chr8(+)	13165845	13165967	13165851	13165967	13159160	13159434	6.10E-05	-0.201
Asb10	chr5(-)	24533650	24533798	24533650	24533794	24534106	24534220	7.48E-05	-0.259
Cbx7	chr15(-)	79918752	79919104	79918752	79918927	79921371	79921438	1.03E-05	-0.278

Nptn	chr9(+)	58650496	58650518	58650508	58650518	58643528	58643802	3.02E-08	-0.352
Atp2a2	chr5(-)	122456454	122456617	122456454	122456570	122457302	122457426	4.37E-11	-0.373

A5SS

gene	chr	longExonStart	longExonEnd	shortES	shortEE	flankingES	flankingEE	P-Value	IncLevelDiffer
Mia3	chr1(-)	183329585	183329819	183329745	183329819	183328203	183328345	5.66214E-15	0.449
Lrrfip2	chr9(+)	111179063	111179221	111179063	111179097	111179872	111179920	4.65739E-13	0.438
Chd9	chr8(+)	91036325	91036598	91036325	91036550	91037406	91037575	1.14338E-05	0.538
Tnk2	chr16(+)	32678668	32678785	32678668	32678713	32679458	32680807	1.31535E-05	0.412
Tacc2	chr7(+)	130734948	130735243	130734948	130735150	130739811	130739873	7.77156E-16	0.248
Snhg5	chr9(-)	88521917	88522381	88522306	88522381	88521649	88521716	1.10878E-05	-0.308

RI

gene	chr	riExonStart	riExonEnd	upstreamES	upstreamEE	downstreamES	downstreamEE	P-Value	IncLevelDiffer
Zfp326	chr5(+)	105886316	105888839	105886316	105886428	105888433	105888839	1.44915E-05	0.637
Lyl1	chr8(+)	84702639	84702995	84702639	84702690	84702790	84702995	0.000191645	0.292
Galt	chr4(+)	41756512	41756995	41756512	41756561	41756938	41756995	0.000142222	0.253
Myzap	chr9(-)	71552238	71555737	71552238	71552364	71555584	71555737	0.000215629	0.229
Isyna1	chr8(+)	70595138	70595554	70595138	70595271	70595360	70595554	0.00023002	0.226
Neu2	chr1(+)	87594549	87595089	87594549	87594617	87594877	87595089	0.000558379	-0.401
Rad52	chr6(+)	119918586	119919077	119918586	119918786	119918925	119919077	4.41186E-07	-0.462

MXE

gene	chr	1stExonStart	1stExonEnd	2ndExonStart	2ndExonEnd	upstreamES	upstreamEE	downstreamES	downstreamEE	P-Value	IncLevelDiffer
Lrrfip1	chr1(+)	91082140	91082179	91084945	91085005	91079030	91079081	91088633	91088711	0	0.685
Abcd3	chr3(-)	121786001	121786071	121787979	121788049	121784472	121784570	121791733	121791822	0	0.603
Lrrfip1	chr1(+)	91076943	91076991	91079030	91079081	91073323	91073341	91084945	91085005	1.53E-12	0.344
Lrrfip1	chr1(+)	91084945	91085005	91088633	91088711	91082140	91082179	91091601	91091667	8.35E-08	0.29
Tmem234	chr4(+)	129601406	129601533	129601869	129601961	129600982	129601243	129602194	129602261	0.000558288	0.267
Neb	chr2(-)	52160655	52160748	52161370	52161475	52151363	52151456	52162067	52162172	0	0.238
Lrrfip2	chr9(+)	111179176	111179221	111179872	111179920	111179046	111179097	111180215	111180272	0.000422588	0.223
Tnnt3	chr7(+)	142513664	142513705	142514326	142514366	142512531	142512622	142515775	142516003	4.02E-08	0.206
Reep1	chr6(+)	71799165	71799201	71805342	71805494	71795146	71795324	71807790	71810246	4.83E-09	-0.228
Slc8a3	chr12(-)	81209453	81209471	81214065	81214169	81204891	81205016	81314259	81316104	0.00000631	-0.235
Neb	chr2(-)	52161370	52161475	52162067	52162171	52160655	52160748	52162904	52163009	0	-0.246
Pdlim5	chr3(-)	142306283	142306298	142312122	142312214	142304704	142304722	142314405	142314448	0	-0.403
Pdlim5	chr3(-)	142309495	142309544	142312122	142312214	142306283	142306298	142314405	142314448	4.44E-16	-0.469
Atp11a	chr8(+)	12861639	12861724	12863758	12863868	12859313	12859479	12864919	12868667	0.000000357	-0.565
Lrrfip1	chr1(+)	91082140	91082179	91084945	91085005	91079030	91079081	91093559	91093637	0	0.84
Lrrfip1	chr1(+)	91084945	91085005	91093559	91093637	91082140	91082179	91100576	91100612	0	0.354
Lrrfip1	chr1(+)	91082140	91082179	91091601	91091667	91079030	91079081	91093559	91093637	0	0.345
Phkb	chr8(+)	86025197	86025288	86026456	86026547	86024280	86024338	86029481	86029684	8.11129E-12	0.338
Clk4	chr11(+)	51268768	51268846	51270536	51270595	51268164	51268231	51271799	51272022	0.001014603	0.304
Arhgap12	chr18(-)	6069830	6070091	6111684	6112439	6064340	6064421	6115813	6115850	3.97665E-05	0.284

Lrrfip1	chr1(+)	91087058	91087124	91088633	91088762	91084945	91085005	91091601	91091667	2.20981E-07	0.277
Rab7	chr6(-)	88015631	88015718	88038809	88038880	88013637	88013698	88045072	88045231	5.64223E-06	0.264
Nme6	chr9(+)	109839607	109839710	109841475	109841515	109833195	109833295	109841926	109842087	0.0020038	0.24
Slc25a3	chr10(-)	91121921	91122043	91122206	91122331	91119526	91119706	91123557	91123706	0	0.208
Mef2d	chr3(+)	88158033	88158171	88158220	88158355	88156321	88156525	88159121	88159332	6.03951E-06	-0.153
Nt5c2	chr19(-)	46898802	46898894	46924261	46924335	46898621	46898679	46950845	46950965	0.001618525	-0.215
Ldb3	chr14(-)	34572989	34573012	34576985	34577344	34567397	34567567	34578594	34578670	0	-0.238
Pacsin3	chr2(+)	91256301	91256408	91256889	91256926	91256143	91256190	91259863	91259927	2.63363E-05	-0.249
Haus7	chrX(-)	73448810	73448902	73452998	73453060	73448191	73448350	73454866	73454934	0.000789618	-0.297
Rbpms	chr8(-)	33806716	33806850	33834303	33834454	33795081	33795150	33862985	33863048	0.001709407	-0.321
Arl2bp	chr8(+)	94667596	94667658	94670320	94670427	94666599	94666901	94671294	94671380	1.0522E-09	-0.34
D630024D03Rik	chr11(-)	31802829	31802981	31804035	31804197	31802331	31802448	31824292	31824524	0.00118454	-0.341
Cbwd1	chr19(-)	24953259	24953351	24955335	24955432	24949201	24949261	24957903	24957990	2.5156E-05	-0.343
Lrrfip1	chr1(+)	91084945	91085005	91091601	91091667	91079030	91079081	91093559	91093637	0	-0.396

column-index

chr: chromosome

+/-: plus / minus strand

exonStart: Start position of alternative exon in chromosome

exonEnd: End position of alternative exon in chromosome

upstreamES: Constitutive up-stream exon start site in chromosome

upstreamEE: Constitutive up-stream exon end site in chromosome

downstreamES: Constitutive down-stream exon start site in chromosome

downstreamEE: Constitutive down-stream exon end site in chromosome

IncLevelDiffer: Inclusion level difference

SE: Skipped exon

A3SS: Alternative 3’ splice site

A5SS: Alternative 5’ splice site

RI: Retained intron

MXE: Mutually exclusive exon

Table S4. Rbm24-regulated AS events associated with myogenic differentiation.

gene symbol	splicing type	alternative exon loci	C2C12 Differ ΔPSI#	UKO mice ΔIncLevelDiffer#
Ppfibp1	SE	chr7(+): 146996808-146996901	0.42	0.63
R3hdm1	SE	chr1(+): 128162768-128162858	0.22	0.59
Cad	SE	chr5(+): 31075470-31075521	0.2	0.57
Uso1	SE	chr5(+): 92183481-92183502	0.63	0.54
Cog1	SE	chr11(+): 113661658-113661693	0.2	0.47
Ranbp2	SE	chr10(+): 58481318-58481396	0.31	0.42
Ppp3ca	SE	chr3(+): 136932010-136932040	0.38	0.32
Tmed2	SE	chr5(+): 124543486-124543507	0.42	0.37
Naca	SE	chr10(+): 128039170-128045086	0.31	0.37
R3hdm2	SE	chr10(+): 127465130-127465184	0.23	0.35
Lpin1	SE	chr12(-): 16571163-16571262	0.2	0.29
Ssr1	SE	chr13(-): 37983094-37983109	0.23	0.31
Stim2	SE	chr5(+): 54110115-54110139	0.27	0.3
Csde1	SE	chr3(+): 103036574-103036721	0.32	0.3
Arfgap2	SE	chr2(+): 91269069-91269111	0.38	0.29
Cltc	SE	chr11(-): 86701060-86701081	0.24	0.21
Tpm3	SE	chr3(+): 90091012-90091091	0.46	0.29
Prpf18	SE	chr2(-): 4648096-4648123	0.2	0.29
Rnps1	SE	chr17(+): 24418037-24418228	0.2	0.27
Psma4	SE	chr9(+): 54951343-54951428	0.23	0.26
Hnrnph2	SE	chrX(+): 134602917-134602962	0.31	0.25
Tacc2	SE	chr7(+): 130744526-130744616	0.32	0.23
Sema6c	SE	chr3(+): 95171790-95171886	0.41	0.21
Upf3b	SE	chrx(-): 37099065-37099104	0.22	0.21
Tnk2	A3SS	chr16(+): 32678668-32678785	0.35	0.41
Ubr3	SE	chr2(+): 69971401-69971434	0.64	0.53
Fxr1	SE	chr3(+): 34064973-34065054; 34068160-34068252	0.82	0.26
Txnl1	SE	chr18(-): 63665651-63665694	0.3	0.41
Lrrfip1	SE	chr1(+): 91108579-91108672; 91110354 -91110447	0.48	0.19
Lrrfip2	SE	chr9(+): 111179176-111179221	0.46	0.44
Coro6	SE	chr11(+): 77467749-77467869	0.78	0.35
Itga7	SE	chr10(+): 128956610-128956723	0.27	0.25
Rock2	SE	chr12(+): 16973439-16973610	0.44	0.39
Sorbs1	SE	chr19(-): 40361428-40361503	0.53	0.42
Taf12	SE	chr4(+): 132281993-132282078	0.33	0.25
Mef2d	MXE	chr3(+): 88158033-88158171; 88158220-88158355	0.47	0.15
Ppp1r12a	SE	chr10(+): 108248955-108249069	0.43	0.49

Cobl	SE	chr11(-): 12373328-12373373	0.53	0.23
Usp28	SE	chr9(+): 49032508-49032688	0.62	0.22
Kif13a	SE	chr13(-): 46808156-46808276	0.23	0.28
Palld	SE	chr8(-): 61514987-61515034	0.24	0.24
Phtf2	SE	chr5(-): 20812940-20812993	0.39	0.21
Usp25	SE	chr16(+): 77097648-77097762	0.33	0.28
Bnip3l	SE	chr14(-): 66988885-66988941	0.37	0.2
Pdlim5	A3SS	chr3(-): 142312122-142312541	0.23	0.64
Slc25a3	MXE	chr10(-): 91121921-91122043; 91122206-91122331	0.57	0.21
Pdlim3	SE	chr8(+): 45908468-45908656	0.52	0.2

Legend: Genes abnormally spliced in skeletal muslcle in response to deletion of Rbm24 are compared with genes alternatively spliced during myoblast differentiation to identify potential alternative splicing events which are involved in myogenic differentiation regulation. The loci of alternative exons in genome (GRCm38/mm10) are shown. Abbreviations: A3SS, alternative 3' splice site; MXE, mutually exclusive exon; SE, skipped exon. C2C12 Differ ∆PSI[#]: Inclusion level difference (absolute value) of each alternative exon during C2C12 differentiation. PSI, percent spliced in. UKO mice ∆IncLevelDiffer[#]: Inclusion level difference (absolute value) of each alternative exon in skeletal muscle of Rbm24 knockout mice.

Table S5. Rbm24 and Rbfox2 regulate a common set of AS events.

Gene Symbol	Splicing Type	Alternative cassette loci	Rbfox2 KD ΔPSI[#]	Rbm24 KO ΔIncLevel Difference[#]
Ubr3	SE	chr2(+): 69971401-69971434	0.24	0.53
Fxr1	SE	chr3(+): 34064973-34065054; 34068160-34068252	0.33	0.25
Txnl1	SE	chr18(-): 63665651-63665694	0.28	0.41
Lrrfip1	SE	chr1(+): 91108579-91108672; 91110354 -91110447	0.23	0.24
Coro6	SE	chr11(+): 77467749-77467869	0.21	0.26
Itga7	SE	chr10(+):128956610-128956723	0.26	0.25
Rock2	SE	chr12(+):16973439-16973610	0.4	0.39
Sorbs1	SE	chr19(-): 40361041-40361104	0.44	0.48
Ccdc85c	SE	chr12(-): 108216786-108216825	0.22	0.23
Clasp1	SE	chr1(+): 118547655-118547658	0.39	0.47
Ranbp2	SE	chr10(+): 58481318-58481396	0.24	0.42
Taf12	SE	chr4(+):132281993-132282078	0.31	0.25
Mef2d	MXE	chr3(+): 88158033-88158171; 88158220-88158355	0.39	0.15
Cobl	SE	chr11(-): 12373328-12373373	0.50	0.23
Usp28	SE	chr9(+): 49032508-49032688	0.45	0.32
Kif13a	SE	chr13(-): 46808156-46808276	0.22	0.28
Palld	SE	chr8(-): 61514987-61515034	0.23	0.24
Phtf2	SE	chr5(-): 20812940-20812993	0.39	0.21
Usp25	SE	chr16(+): 77097648-77097762	0.26	0.28
Bnip3l	SE	chr14(-): 66988899-66988941	0.31	0.20
Pdlim5	A3SS	chr3(-): 142312122-142312541	0.21	0.35
Slc25a3	MXE	chr10(-): 91121921-91122043; 91122206-91122331	0.23	0.21
Pdlim3	SE	chr8(+): 45908468-45908656	0.35	0.20

#: the absolute value of ΔPSI or ΔIncLevelDifference.

Legend: We compared AS events in response to Rbm24 in adult skeletal muscle with AS events in response to Rbfox2 during myogenesis (C2C12 differentiation). In total, 23 AS events were identified to be regulated by both Rbm24 and Rbfox2. Abbreviations: AS: alternative splicing; SE: skipped exons; MXE: mutually exclusive exons. PSI: percent spliced in; IncLevelDifference: inclusion level difference.

Table S6. Primer list.

Clone primer	Sequence 5'-3'
Lrrfip1 Mini-F-*Kpn*I	acttaagcttggtaccGACAGAAATATGCCACCGAGCT
Lrrfip1 Mini-R-*Xba*I	gtttaaacgggccctctagaCTAGGGTCCCCTCCCCGGCCGA
Mef2d Mini-F-*Kpn*I	acttaagcttggtaccGATTTCCATCAACCGTGGCTA
Mef2d Mini-R-*Xba*I	gtttaaacgggccctctagaTACACATACAGAAACGCTGCAA
Rock2 Mini-F-*Kpn*I	acttaagcttggtaccAATTATTTTCTTATCTTTGCAG
Rock2 Mini-R-*Xba*I	gtttaaacgggccctctagaTGTCTGAAAGTATAGGCATGTC
Plvcs-Mef2d-F-*Xba*I	ctagtctagaATGGGGAGGAAAAAGATTCAGAT
Plvcs-Mef2d-R-*Mlu*I	cgacgcgtTCACTTTAATGTCCAAGTATCCAG
Pxj40-Rbm24-F-*Xho*I	ctcgagATGCACACGACCCAGAAGGAC
Pxj40-Rbm24-R-*Kpn*I	ggtaccCTATTGCATTCGGTCTGTCTGC

shRNA primer	Sequence 5'-3'
Pll3.7-mshRbm24-F-2014	TagctgctgcaggctatgtaacTTCAAGAGA gttacatagcctgcagcagctTTTTTTC
Pll3.7-mshRbm24-R-2014	TCGAGAAAAAAagctgctgcaggctatgtaacTCTCTTGAAgttacatagcctgcagcagctA
Pll3.7-mshCTL-F	TgtagcgcggtgtattatacTTCAAGAGAgtataatacaccgcgctacTTTTTTC
Pll3.7-mshCTL-F	TCGAGAAAAAAgtagcgcggtgtattatacTCTCTTGAAgtataatacaccgcgctacA

qPCR primer	Sequence 5'-3'
cTnT-F	GCAGAAGAGGTTGGTCCTGATG
cTnT-R	CACCAAGTTGGGCATGAAGAG
Tmem8c-F	ATCGCTACCAAGAGGCGTT
Tmem8c-R	CACAGCACAGACAAACCAGG
eMHC-F	TCTAGCCGGATGGTGGTCC
eMHC-R	GAATTGTCAGGAGCCACGAAA
Mmp13-F	TTTGAGAACACGGGGAAGA
Mmp13-R	ACTTTGTTGCCAATTCCAGG
Col12a1-F	ATACCATACAAAGGCGGCAAC
Col12a1-R	CGTCCGTGATAATAATTGCCACT
Pax7-F	TGGGGTCTTCATCAACGGTC
Pax7-R	ATCGGCACAGAATCTTGGAGA
Myog-F	GCAGGCTCAAGAAAGTGAATGA
Myog-R	TAGGCGCTCAATGTACTGGAT
Myod1-F	ATGGATTACAGCGGCCCC
Myod1-R	TGTGGAGATGCGCTCCACTA
GAPDH-F	CAATCTGTCCGTCGTGGATC
GAPDH-R	CCTGCTTCACCACCTTCTTG
Rbm24-F	ACTTGGGAGCAAAACCAAGA
Rbm24-R	GAAGCTGTTGAACGCCAAA
Rbfox2-F	ACAACAACTCCTGACGCAAT
Rbfox2-R	GCTACTCTGTTCTCCATGAGGC
hRbm24-F	CCAAGGATCATGCAACCAG

hRbm24-R	GCAGGTATCCCGAAAGGTCT
hHprt-F	CTGAGGATTTGGAAAGGGTG
hHprt-R	TTATGTCCCCTGTTGACTGG
Ppp3ca-constitutive-F	CTGTATGGATGCCTTCGACT
Ppp3ca-constitutive-R	AGCCTCTGACTGTGTTGTGA
Prkca-constitutive-F	CAAATGGGCTTTCGGATCCTT
Prkca-constitutive-R	CCGGCATCTTCATTAGCTCT
Itgb1-constitutive-F	GCTCCCACTTCAATCTCACC
Itgb1-constitutive-R	AAGAACAATTCCAGCAACCAC
Pdlim5-constitutive-F	ATTTAACTGTGCTCATTGCAAA
Pdlim5-constitutive-R	ATCATGCCAAGTGTATCCCA
Rock2-constitutive-F	GGCATCACAGAAGGTTTATGCAA
Rock2-constitutive-R	ACATCTCTGTGTATTAAGCCCAT
Naca-constitutive-F	TCAGTACCAGAGCTCGAGGA
Naca-constitutive-R	TGCCTTCTTCTCACTTCGACT
Mef2d-constitutive-F	TGACATCATCCCTTACGGACCC
Mef2d-constitutive-R	ATGACCCGCAGATCAGGC
Lrrfip1-constitutive-F	CCAGCTAGACAATGAGAAGACCAA
Lrrfip1-constitutive-R	TCTTTCACTTCGGCAAACTGGA

**Alternative splicing primer**	**Sequence 5'-3'**
Pdlim5-F	CCAAGAGTGAGCCGGTT
Pdlim5-R	TGTCCTCAATCAGCCGTTT
Itga7-F	CCTTGCTGGTACTGCT
Itga7-R	CCATATCTCAACGTGATGC
Clasp2-F	GAGCCAAGCTTTCTACACC
Clasp2-R	GACTCGTTGAGCACCA
Sorbs1-F	TTGTACCAGTCTTTCGATCGC
Sorbs1-R	AATATCGAGGGGAATGGACATCA
Rmnd1-F	GTTTCTGAGACACCCGACT
Rmnd1-R	AGTGGTCTTAATGTAAGCCAA
Cog1-F	CTGAGCATGACAAGCACAA
Cog1-R	AAGCTTGAATAATGAAGGTGCAG
Tmed2-F	GCTCCCAAAGGACAAGACA
Tmed2-R	CATGTACTCCTGTTCGTGCT
Cltc-F	ATAAATTAGATGCTTCGGAGT
Cltc-R	AACCATAACCAAAAGGTGC
Tmem65-F	GCGACTTCATCTACAGCC
Tmem65-R	CCATATCTCAGCTGTCCT
Lffrip1-F	TCCTGCAGTTCCAGTTTGCC
Lffrip1-R	CTTTTGTTATCTTCGTAGGGG
Ash2l-F	CCATTTAGGTTCCCACTAGCAG
Ash2l-R	AGGAGCCAAGCGTAAGCA
Txnl1-F	GTACTCCAGTCCAGGCAACA

Txnl1-R	AGTTTCCGAAGAGAATCAAGCA
Psma4-F	TGCTTGGCCATCCGGGTT
Psma4-R	CTGGAGAAAATATTGTGGTCCT
Ubr3-F	CACGAGCTCTGACGCTTT
Ubr3-R	AACTTCTATGTCATCAAGCCAT
Tmed2-F	GCTCCCAAAGGACAAGACA
Tmed2-R	CATGTACTCCTGTTCGTGCT
Cltc-F	ATAAATTAGATGCTTCGGAGT
Cltc-R	AACCATAACCAAAAGGTGC
Tmem65-F	GCGACTTCATCTACAGCC
Tmem65-R	CCATATCTCAGCTGTCCT
Lpin1-F	ATCGTATCCCAGTTCGGACA
Lpin1-R	TTATTCTTTGGCGTCAACCTG
Stim2-F	TGTCCCTGACGCACTACAGA
Stim2-R	ATCTTGTGGTCTACTTCGTCCA
Ryr1-F	CTGGACCTCAATGCCTTCG
Ryr1-R	CCATTGTTGTGCCCCTCG
Trdn-F	AAAATGAGAAAACCTACAGA
Trdn-R	TGCTTTTTTGAGTTTTTCAC
Fxr1-F	ACCACAGTACTAACCGTCGT
Fxr1-R	AGTTGGACCATTTATTGCCTT
Ank2-F	ATGTTCCTGAGACAATGACGGAG
Ank2-R	CCGAAGGTAGTTCATGCCAT
Lrrfip1-F	TTCTGACGAAGACGAGCGCTTG
Lrrfip1-R	TGCCAGATCTTCTACTTAGGGAC
R3hdm1-F	AAAACTATGGGAAGATTTCGG
R3hdm1-R	GTTTCATCTTTTGAGGGCTT
Coro6-F	GAGCCTGGACGACATACACC
Coro6-R	GCCACACAAGTAGACGATGC
Ppp1r12a-F	TACTTCCAGCACGCAGGCA
Ppp1r12a-R	TCTTTCGAAGCCCTAACCTCC
Rock2-F	CATTGAACAGCTGCGGTCAC
Rock2-R	GCACAGGCAATGACAACCA
Ranbp2-F	ATCTTCTGATGGCAAAGTCAA
Ranbp2-R	GCCAGGCCTATTCTTATAACAAA
R3hdm2-F	GGCCTTCTTCCTTGTACTGC
R3hdm2-R	CGGCTAAGGCTCATTTGTCC
Ppp3cb-F	ACAGAAGGTGAAGACCAGTT
Ppp3cb-R	CTTGCCAATTGCTCGGAT
Ppp3ca-F	CCTGACCCCAACTGGCAT
Ppp3ca-R	CATCCTCTCGTTAATTCGGTCT
Prkca-F	TGCCTTCTTCAGGAGAATCG
Prkca-R	TTTTGAGTTTCATACTGCACT
Hprt-F	TGATGAACCAGGTTATGACC

Hprt-R	GTTGAGAGATCATCTCCACC
Mef2d-1-F	TGACCTTCACCAAGCGGAA
Mef2d-1-R	TGTCACAGCCGTTGAAACCC
Mef2d-2-F	TGACCTTCACCAAGCGGAA
Mef2d-2-R	ATCATCTTATCAAACTCCTCGTCT
Cobl-F	AACTACCTGAGAACGCAGA
Cobl-R	CTGTCTCATCATTGCCAAG
Bnip3l-F	AGACACCCTAAACGTGCT
Bnip3l-R	CTTCACAGGCCACACGAA
Usp25-F	AGCAGCCATCAAGAAGTGAC
Usp25-R	GTTTGGCTTGGGCAACTTTC
Usp28-F	TCCGATGCTTGTCTTCTGAG
Usp28-R	GACAAGAACATGCTGAAGGC

**Genotyping primer**	**Sequence 5'-3'**
Mdx-F	AACTCATCAAATATGCGTGTTAGTG
Mdx-mutant-R	GTCACTCAGATAGTTGAAGCCATTTAA
Mdx-wt-R	GTCACTCAGATAGTTGAAGCCATTTAG
Rbm24-FRT-TF2	CATGGATGTTGGTGGTGCTGTC
Rbm24-FRT-TR2	GACCTGGCGGTAGACAGACATTG
Cre-F	ATTTGCCTGCATTACCGGTC
Cre-R	ATCAACGTTTTCTTTTCGG

Table S7. Annotation of alternative exon loci in each gel image in Figures (6E, 7B, 7C, 7D, 7F, S3B and S4B, respectively).

gene symbol	chr	strand	alternative exon loci
Ppp3ca	chr3	+	136932011-136932040
Prkca	chr11	-	107945556-107945604
Rock2	chr12	+	16982650-16982747
Itgb1	chr8	+	128726982-128727049
Pdlim5	chr3	-	142306284-142306298; 142312122-142312541
Tmed2	chr5	+	124543486-124543507
Cltc	chr11	-	86696175-86696213
Ranbp2	chr10	+	58481318-58481396
Mef2d	chr3	+	88158033-88158171; 88158220-88158355
Naca	chr10	+	128039170-128045086
Lrrfip1	chr1	+	91108579-91108672; 91110354-91110447
Fxr1	chr3	+	34064973-34065054; 34068160-34068252
Itga7	chr10	+	128956610-128956723
Ppp1r12a	chr10	+	108248955-108249069
Sorbs1	chr19	-	40361041-40361104
Clasp2	chr9	+	113862436-113862462
Usp25	chr16	+	77097648-77097762
Usp28	chr9	+	49032508-49032688
Cog1	chr11	+	113661658-113661693
Ubr3	chr2	+	69971401-69971434
Tmem65	chr15	+	58807077-58807158

Legend: The loci of alternative exons in each Figure are annotated. Mouse genome, GRCm38/mm10, is referred.

Figure S1

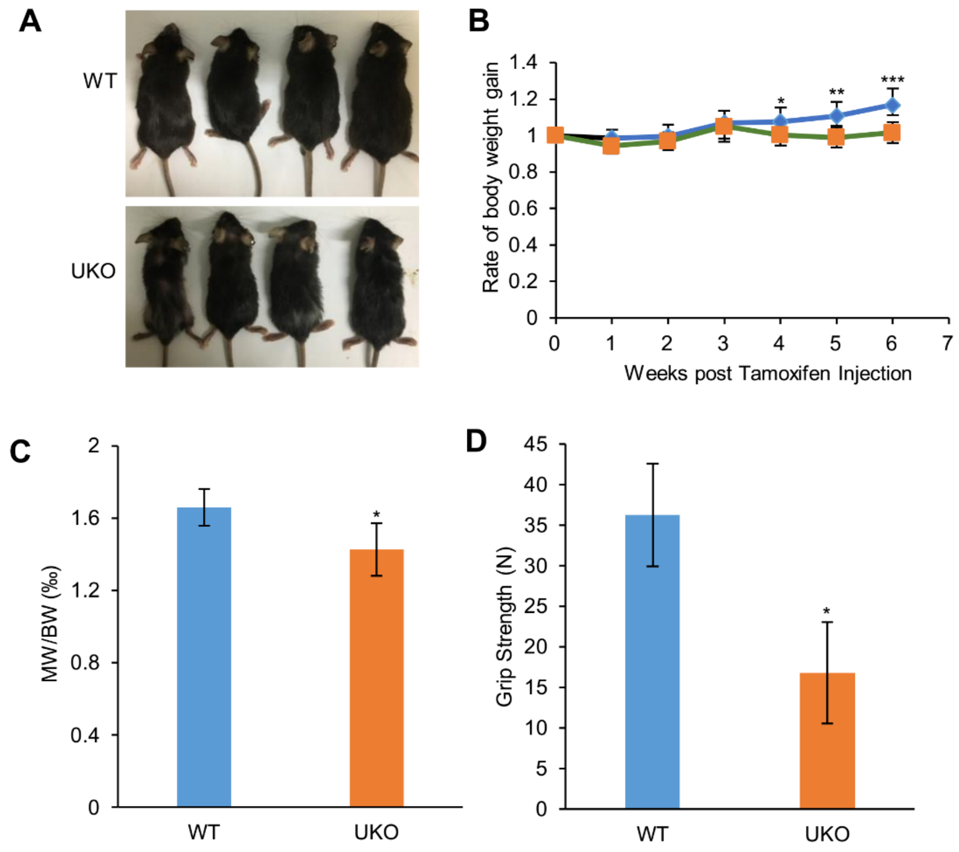

Figure S1. Inducible deletion of Rbm24 leads to a loss of muscle mass and strength, related to Figure 1.

(A) Visual comparison of the body sizes between the WT and UKO mice 1 month after tamoxifen injection. UKO mice showed leaner and smaller body sizes compared with the WT littermates. (B) Statistical analysis of BW. The 8-week-old male mice were administrated tamoxifen to induce Rbm24 knockout, and body weight was examined every week. At 4 weeks after tamoxifen administration, the BW began to decrease significantly in the UKO mice. The BW was normalized to the original weight prior to tamoxifen treatment. Data are presented as mean ± SEM, n = 14-16, *$P < 0.05$, **$P < 0.01$, ***$P < 0.001$, unpaired t-test. (C) Statistical analysis of TA MW. Approximately 2 months after tamoxifen injection, TA MW was significantly reduced in the UKO mice. The TA MW was normalized to BW. Data are presented as mean ± SEM, n = 8, *$P < 0.05$, unpaired t-test. (D) Muscle strength assay. Muscle strength was assessed using an inverted cage flip test. Muscle strength is expressed in terms of physical impulse which is calculated as time (s) ∗ body mass (g) ∗ 0.00980665 N/g. A total of 9 mice in each group were tested in triplicate. Data are presented as mean ± SEM, n = 9, *$P < 0.05$, unpaired t-test. Abbreviations: BW: body weight; MW: muscle weight; TA muscle: tibialis anterior muscle; UKO: UBC-CreERT2, Rbm24$^{loxP/loxP}$.

Figure S2

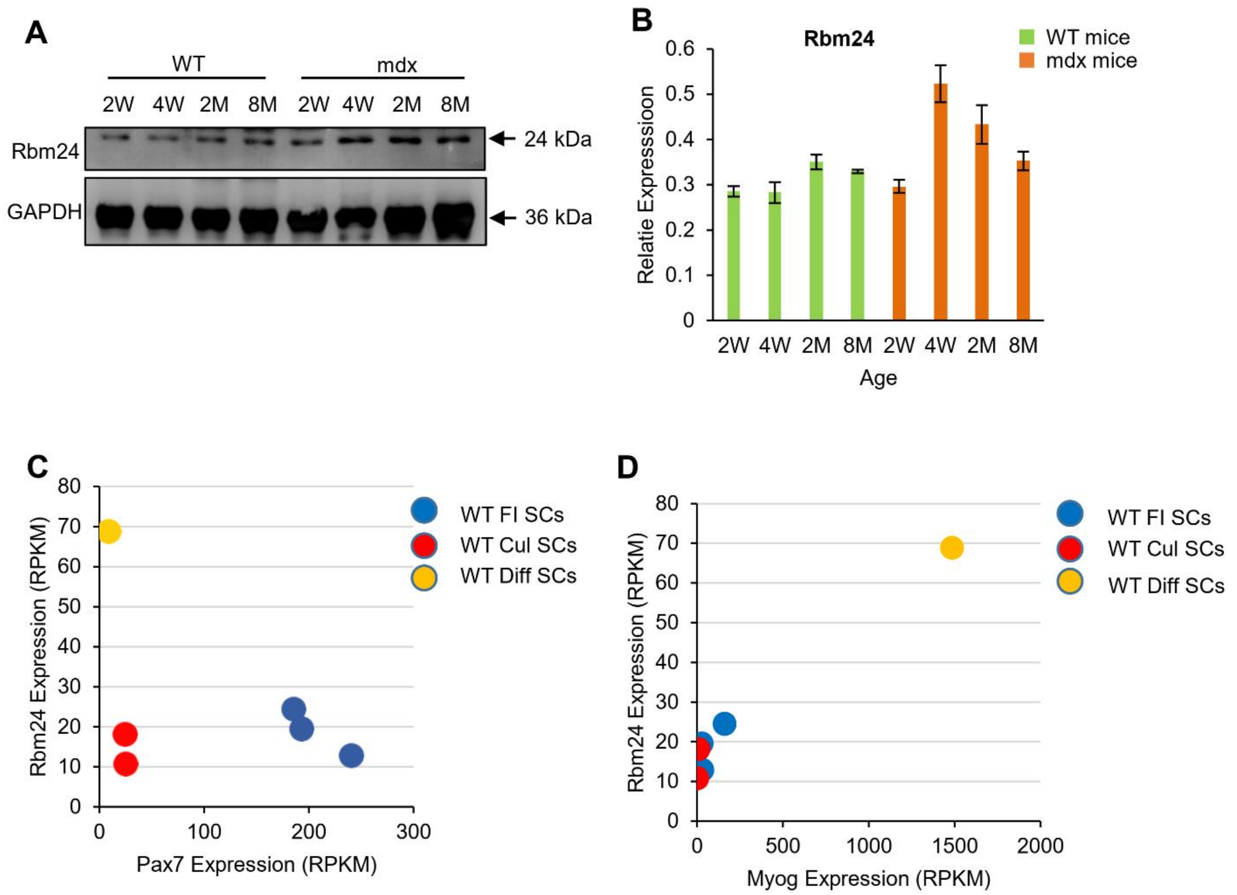

Ryall et al., 2015

Figure S2. Analysis of Rbm24 expression in mdx mice and satellite cells, related to Figure 2.

(A) Rbm24 expression in the TA muscle of mdx mice compared with WT mice of the same age. Dynamic Rbm24 expression was assessed in mdx mice and WT mice (C57BL/6) during development using western blotting. GAPDH was used as the loading control. (B) Histogram showing the expression levels of Rbm24 in (A). Expression of Rbm24 was normalized to GAPDH. Data are presented as mean ± SEM from three independent experiments. (C) Co-expression analysis of Rbm24 with Pax7 in satellite cells of different statuses. (D) Co-expression analysis of Rbm24 with Myogenin in satellite cells of different statuses. Gene expression data were from the RNA-Seq results of mice satellite cells. Gene expression levels are presented as RPKM values. Each point indicates the Rbm24 expression in satellite cells of different statuses, indicated as the differential expression of the marker genes Pax7 or Myogenin. Abbreviations: RPKM: reads per kilobase per million mapped reads; TA muscle: tibialis anterior muscle; WT: wild type; WT FI SCs: wild type freshly isolated satellite cells (quiescent satellite cells); WT Cul SCs: wild type cultured satellite cells (activated satellite cells); WT Diff SCs: wild type differentiated satellite cells (differentiated satellite cells).

Figure S3

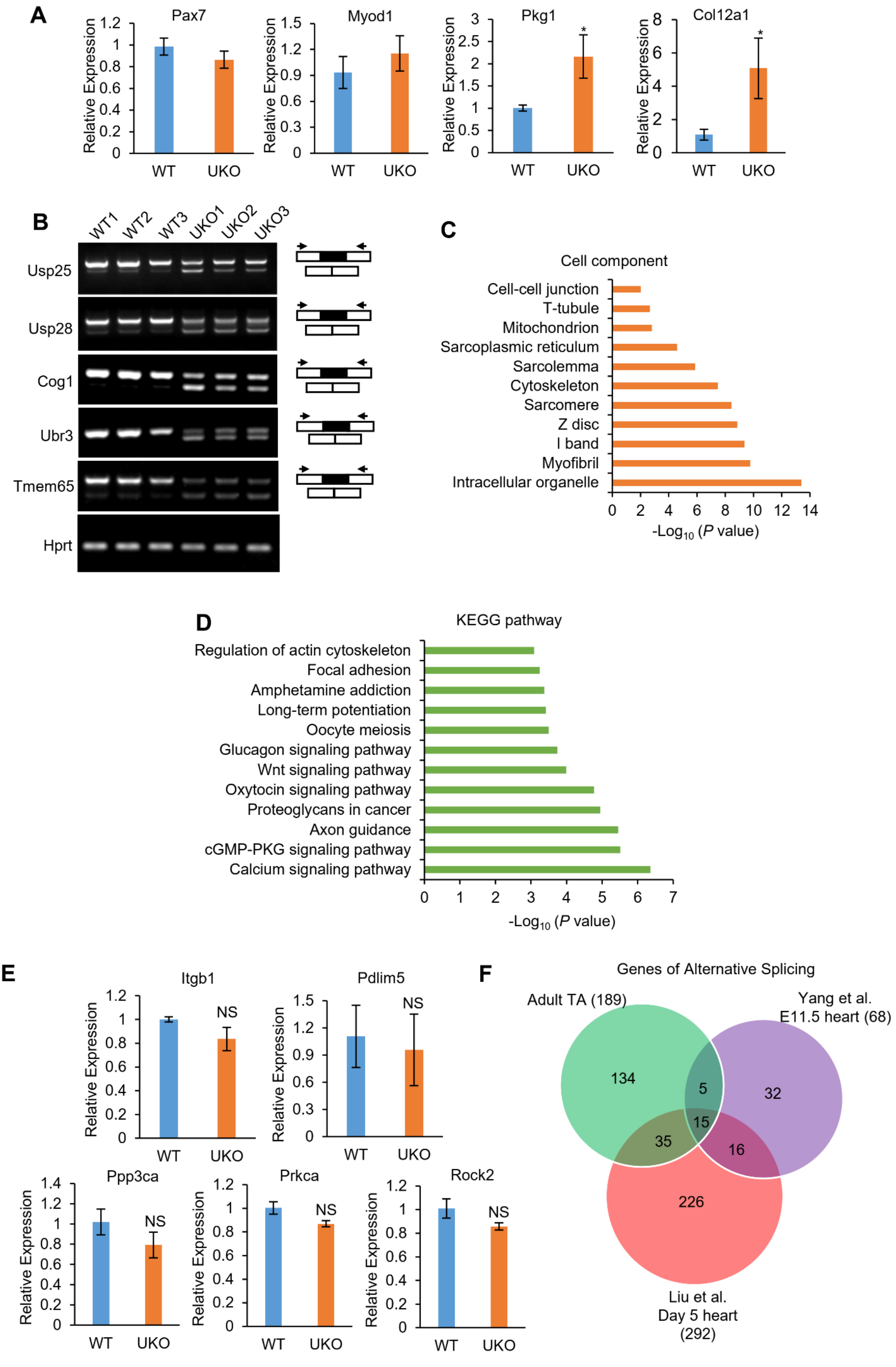
A
Pax7
Myod1
Pkg1
Col12a1
Relative Expression
WT
UKO
*
B
WT1
WT2
WT3
UKO1
UKO2
UKO3
Usp25
Usp28
Cog1
Ubr3
Tmem65
Hprt
C
Cell component
Cell-cell junction
T-tubule
Mitochondrion
Sarcoplasmic reticulum
Sarcolemma
Cytoskeleton
Sarcomere
Z disc
I band
Myofibril
Intracellular organelle
-Log10 (P value)
D
KEGG pathway
Regulation of actin cytoskeleton
Focal adhesion
Amphetamine addiction
Long-term potentiation
Oocyte meiosis
Glucagon signaling pathway
Wnt signaling pathway
Oxytocin signaling pathway
Proteoglycans in cancer
Axon guidance
cGMP-PKG signaling pathway
Calcium signaling pathway
-Log10 (P value)
E
Itgb1
Pdlim5
Ppp3ca
Prkca
Rock2
NS
F
Genes of Alternative Splicing
Adult TA (189)
Yang et al.
E11.5 heart (68)
Liu et al.
Day 5 heart
(292)
134
5
32
15
35
16
226

Figure S3. Analysis of RNA-Seq results, related to Figure 6.

(A) qPCR analysis of differentially expressed genes. Total RNAs were extracted from the TA muscle of 2-month-old mice 3 days after tamoxifen injection, and were reverse transcribed (n = 3 per group). Data are presented as mean ± SEM. **P* < 0.05, unpaired t-test. (B) RT-PCR analysis of AS events based on RNA-Seq. Total RNAs were extracted from the TA muscle of 2-month-old mice 3 days after tamoxifen injection for further AS assay. The primers are indicated by the arrows. White boxes, flanking constitutive exons; gray and black boxes, alternative exons. (C) GO analysis (cell component analysis) showing the enrichment of genes involved in muscle cell structures. (D) KEGG pathway analysis of alternatively spliced genes. Multiple pathways essential for muscle development and muscle function were significantly enriched, such as the calcium signaling pathway and Wnt signaling pathway. (E) qPCR to analyze the expression of Ppp3ca, Prkca, Rock2, Itgb1, and Pdlim5. Total RNAs were extracted from TA muscle of adult mice 3 days after tamoxifen treatment and then reverse transcribed (n = 3 per group). Data are presented as mean ± SEM, NS, not significant. (F) A comparison of the Rbm24-regulated genes in adult TA muscle, E11.5 heart and Day 5 heart. Abbreviations: AS: alternative splicing; Day 5 heart: mice heart at 5 days after birth; E11.5 heart: embryonic heart at 11.5 days post fertilization; GO: Gene Ontology; KEGG: Kyoto Encyclopedia of Genes and Genomes; qRT-PCR: quantitative real-time polymerase chain reaction; TA: tibialis anterior muscle.

Figure S4

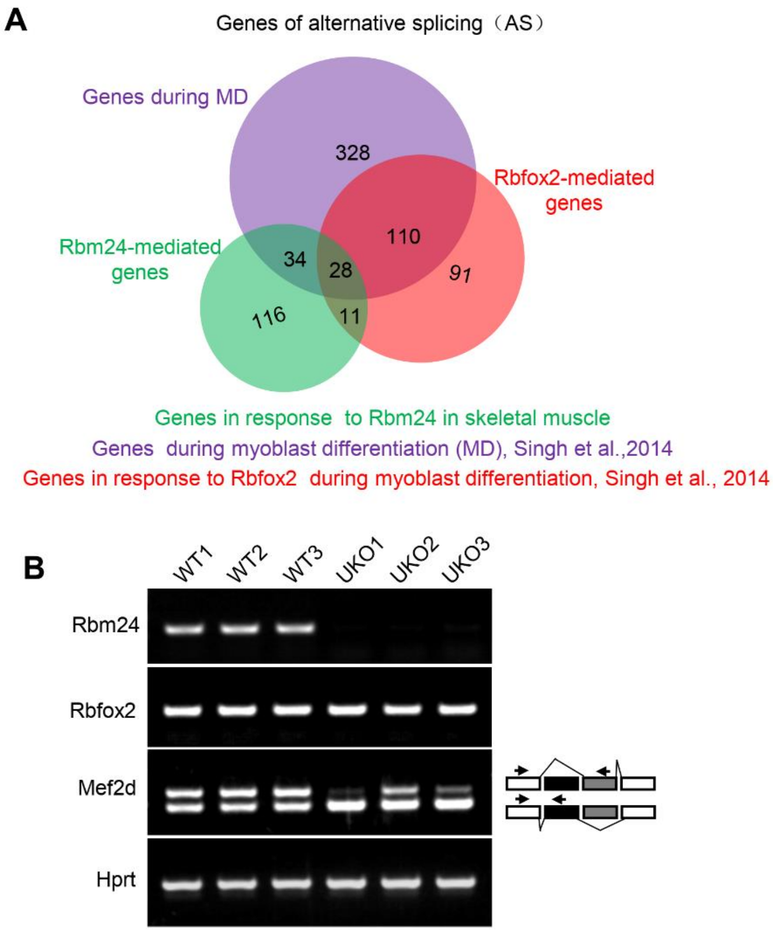

Figure S4. Rbm24 regulates AS events associated with myogenic differentiation, related to Figure 7.

(A) A comparison between genes that were alternatively spliced in the skeletal muscle of Rbm24 knockout mice with genes that were alternatively spliced during C2C12 differentiation with or without Rbfox2 knockdown. There are 62 Rbm24-regulated genes found to be alternatively spliced during C2C12 differentiation. There are 39 genes found to be alternatively spliced in an Rbm24- and Rbfox2-dependent manner. Among these 39 genes, 28 genes were found to be alternatively spliced during myoblast differentiation. (B) RT-PCR analysis of the expression levels of Rbm24 and Rbfox2 and AS of Mef2d in TA muscle of WT and Rbm24 knockout mice. Rbfox2 expression was not affected in Rbm24 knockout mice due to deletion of Rbm24. White box, constitutive exons; gray and black box, alternative exons. The primers used for RT-PCR are indicated by arrows. Hprt was used as loading control. Abbreviations: AS: alternative splicing; RT-PCR: reverse transcription-polymerase chain reaction; TA muscle: tibialis anterior muscle; WT: wild type.
